# Vascular Bundle for Exceptional Water Confinement, Transport, and Evaporation

**DOI:** 10.1021/acsmaterialslett.3c01593

**Published:** 2024-01-12

**Authors:** El Said A. Nouh, Tianyu Liu, Zacary L. Croft, Guoliang Liu

**Affiliations:** ^†^Department of Chemistry, ^‡^Macromolecules Innovation Institute, and ^§^Department of Materials Science and Engineering, Virginia Tech, Blacksburg, Virginia 24061, United States; ∥ Nuclear Materials Authority, P.O. 530, El Maadi, Cairo Egypt

## Abstract

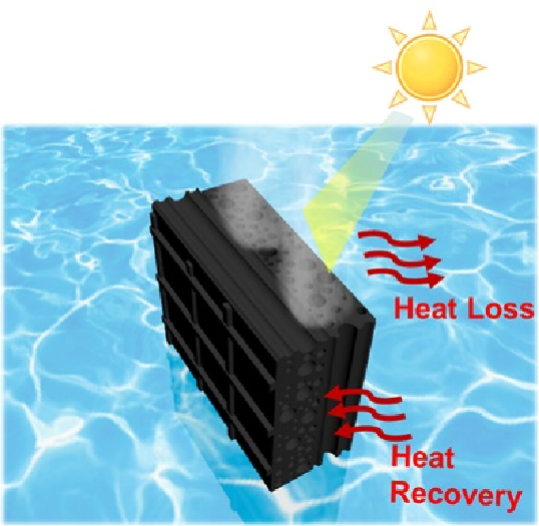

Nature, through billions of years of evolution, has constructed extremely efficient biosystems for transporting, confining, and vaporizing water. Mankind’s ability to master water, however, is far from impeccable, and a sustainable supply of clean fresh water remains a global challenge. Here, we learn from Nature and prepare papyrus carbon (PC) from Egyptian papyrus paper as a sustainable solar desalination material. By taking advantage of the capillary pores from vascular bundles that are inherently built for transporting water in plants, PC achieves an evaporation rate of 4.1 kg m^–2^ h^–1^ in a passive single-stage device. Raman spectroscopy and thermal calorimetry show that the capillary pores pose a confinement effect to generate loosely hydrogen-bonded intermediate water, which substantially reduces the enthalpy of vaporization, allowing for exceptionally high energy efficiencies. The understanding is applicable to all nature-designed vascular plants and man-made separation and purification systems.

One sure thing about understanding and working with water is that we must learn from Mother Nature because she has built so many masterpieces on this water-covered blue planet through billions of years of evolution. Unfortunately, a sustainable supply of clean fresh water remains a global challenge. Clean water scarcity threatens health and security in many regions of the world, especially in Egypt and Africa at large.^1^ On account of the large body of seawater, desalination is considered a promising solution.^2,3^ In light of the low cost of solar irradiation, solar desalination^4−6^ represents an attractive strategy, complementing reverse osmosis,^7^ vacuum-enhanced evaporation–condensation,^8^ and capacitive desalination^9^ that require expensive forms of energy (e.g., pressure or electricity) and have a high environmental impact.^2^ Recently, many materials have shown the capability of solar desalination, including plasmonic nanostructures,^10−14^ polymer gels,^15−19^ porous carbon fibers,^20^ graphene,^21,22^ graphene oxide,^23,24^ graphene/alginate hydrogel,^25^ carbon black,^26^ and carbon foams^27^ in suspension,^10^ floating,^4,28^ or contactless^29^ mode. However, compared to Nature’s uncanny craftsmanship, we must deepen our understanding of water transport, confinement, and vaporization to build systems on par with or better than biological systems.

Papyrus plants (*Cyperus papyrus*) are fast-growing, biosustainable, low-cost, and readily available in the Nile delta region of Egypt. As a “gift of the Nile”, papyrus grows up to 16 feet tall along the river (Figure 1a and Figure S1 in the Supporting Information). Since ca. 4000 B.C., ancient Egyptians have used this plant for making papyrus paper (PP) (Figure 1b), one of the oldest writing materials in existence today.^30^ In fact, “papyrus” is the origin of the word “paper”.

**Figure 1:**
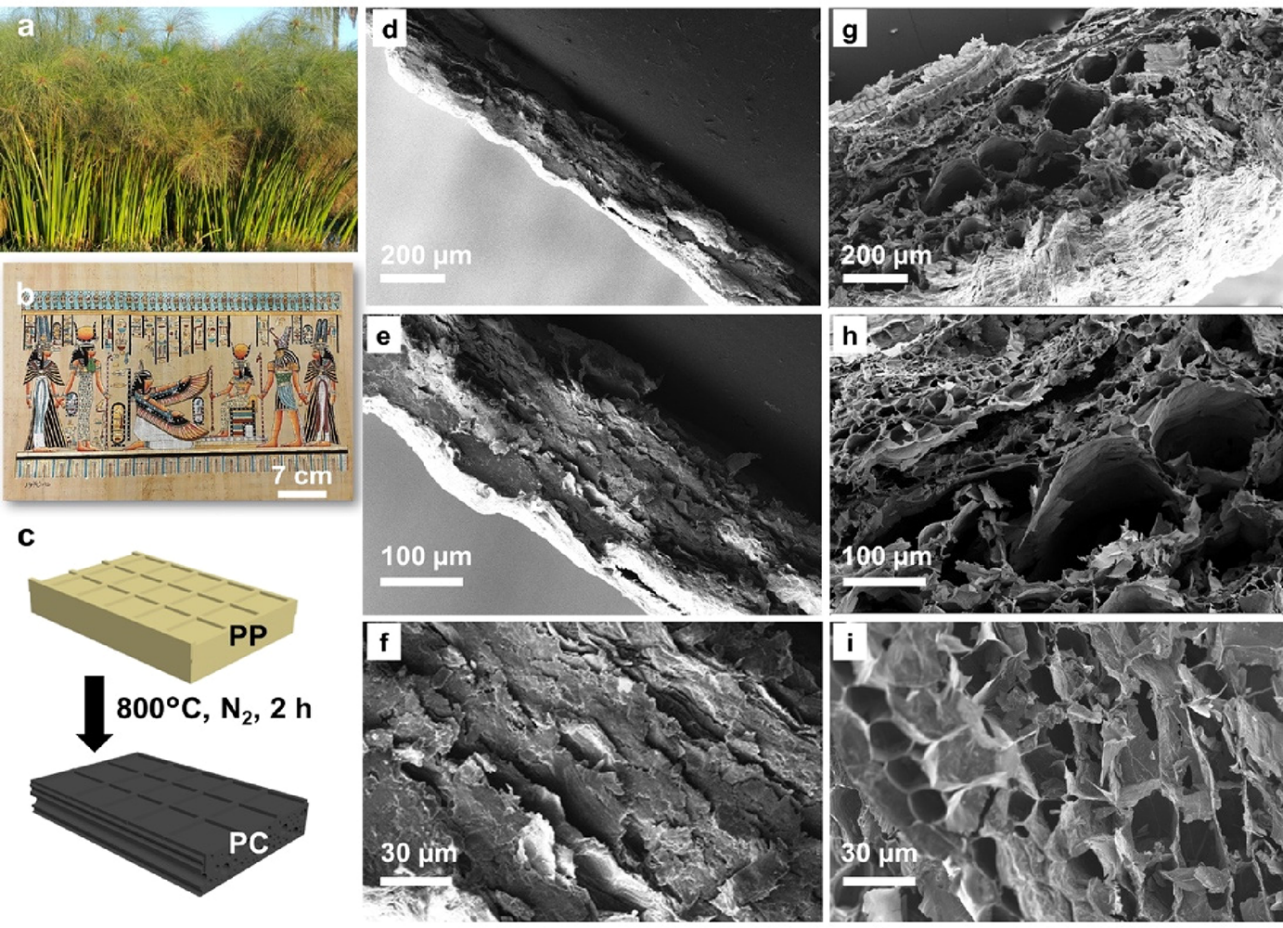
Preparation of PC from PP. (a) Papyrus plant near the Nile River. (b) A piece of papyrus paper made by Egyptian following a 7000-year-old technology. (c) Scheme of the preparation of papyrus carbon from papyrus paper. Cross-sectional SEM images of (d–f) papyrus paper and (g–i) papyrus carbon at different magnifications.

When we evaluate the candidacy of papyrus plants as feedstocks to make solar desalination materials, we find that Nature has provided outstanding structure, properties, and functions through evolution. Growing near water, papyrus has ample vascular bundles to transport water from its root to the top via a capillary effect. In addition, the vascular bundles isolate water within the stem from bulk water, resulting in small pockets of water with a reduced energy loss to the bulk. The as-grown plants, however, cannot efficiently absorb all solar light since they appear green. Thus, we hypothesize that papyrus carbon (PC) after pyrolysis from papyrus paper, if retaining the bundle-like channels for water transport and confinement, is a promising low-cost, scalable, and biosustainable material for solar desalination. Carbonization of plants had been reported previously; however, our design is fundamentally different from the use of natural plant such as bamboo,^31^ mushroom,^32^ or lotus seedpot.^33^ The material that we use here is an industrially produced commodity that has a special design to enable latent heat recovery.

## Results and Discussion

Following the above rationale, we converted PP to PC through a pyrolysis process under a N_2_ atmosphere at 800 °C (Figure 1c). A close examination revealed the internal microstructures of PP and PC. Papyrus plant possessed vascular bundles, a prominent feature as distinguishable by the naked eye (Figure S2 in the Supporting Information). After pyrolysis, these vascular bundles appeared as lines running horizontally on the PC frontside and vertically on the backside (Figure S3 in the Supporting Information). The different vascular bundle orientation is attributed to the manufacturing method of the papyrus sheets^34^ (see the Supporting Information for more details). Both PP and PC revealed a honeycomb-like network derived from parenchyma cells,^30^ as shown by scanning electron microscopy (SEM) micrographs (Figure S4 in the Supporting Information). The honeycomb-like network built up the vascular bundles that run through the length of the entire stem of a papyrus plant (Figure S5 in the Supporting Information). The cross sections of the bundles appear as macropores in the cross-sectional view of the PC (see Figures 1g–i). During the manufacture of papyrus sheets, the macropores were closed due to compression. Therefore, under SEM, the PP sheets revealed only limited slit pores (Figures 1d–f). During pyrolysis, PP expanded its volume, as evidenced by the increased thickness of PC. As a result, the slit pores reopened and became accessible for transporting water, thus enabling solar desalination. The pore reopening was attributed to the carbonization process, which produced ample gaseous products and puffed up the porous network of PC. Notably, the re-expanded vascular bundles extended throughout PC and aligned parallel to its surface. If needed, further PC activation by KOH can generate additional micropores in PC (Figure S6 in the Supporting Information).

**Figure 2:**
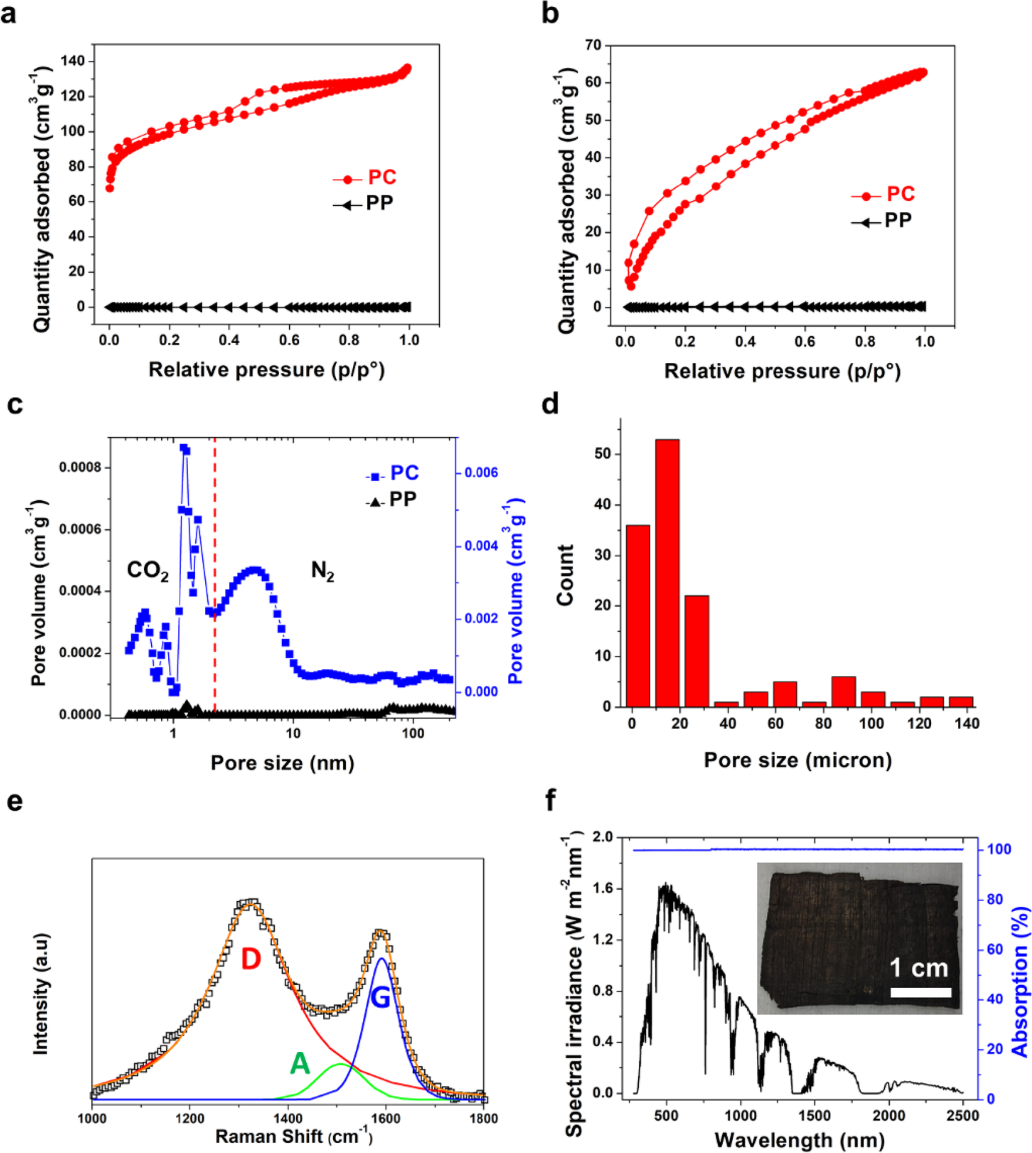
Physical characterizations of PC. (a) N_2_ and (b) CO_2_ physisorption isotherms of PP and PC. (c) Micropore and mesopore size distributions of PP and PC based on N_2_ and CO_2_ isotherms, respectively. (d) Macropore size distributions of PC based on SEM image analysis. (e) Raman spectrum of PC. (f) Light absorption (blue) of PC (inset) in the wavelength range of 200–2500 nm (black).

The surface areas and pore size distributions of PP and PC were evaluated by gas physisorption using N_2_ and CO_2_ (Figures 2a and 2b). PP showed little, if any, adsorption of N_2_ and CO_2_, suggesting that it was mostly nonporous, in agreement with the SEM images. In contrast, PC exhibited type IV and type I isotherms for N_2_ and CO_2_ physisorption, respectively, as defined by the International Union of Pure and Applied Chemistry (IUPAC). The hysteresis loop in the N_2_ physisorption isotherm indicated the presence of mesopores in PC. The mesopore and micropore size distributions were determined using nonlocal density functional theory (NLDFT) based on the N_2_ and CO_2_ isotherms, respectively. PC contained micropores and mesopores with prominent peak pore sizes at ∼1.3 and 4.7 nm (Figure 2c). The drastically different pore size distributions were also reflected in the surface areas. N_2_-based BET surface areas of PP and PC were ∼0.147 and 368 m^2^ g^–1^, respectively. As measured from the cross-sectional SEM images, the macropore size distribution ranged from ∼3 to 140 μm and peaked at ∼15 μm (Figure 2d).

The Raman spectrum of PC (Figure 2e) revealed three prominent peaks, deconvoluted to D, A, and G bands at 1321, 1503, and 1592 cm^–1^, respectively. The intensity ratio between D and G bands (*I*_D_/*I*_G_) was ∼4.5, indicative of a low graphenic degree of PC.^35^ The A band at 1503 cm^–1^, which was associated with sp^3^ carbon atoms between sp^2^ hybridized carbon atoms or point defects,^35^ corroborated with the high *I*_D_/*I*_G_ value of PC.

Optically, PC appeared black, indicative of strong light absorption. The light absorption efficiency was nearly 100% (Figure 2f), as confirmed by the zero reflectance and transmittance across the solar spectrum of 200–2500 nm, meaning that PC effectively absorbed all photons in the solar light. In addition to its blackness, the high absorption efficiency of PC was also attributed to the porous nature and surface roughness, both contributing to an increased optical path length and light scattering.^13^

Fourier transform infrared (FTIR) spectroscopy was employed to determine the chemical structure of PC. The FT-IR spectra of PC (Figure S7 in the Supporting Information) exhibited absorption peaks at 3448 cm^–1^ for −OH stretching and 2919 cm^–1^ for C–H stretching. The peak at 2366 cm^–1^ is due to CO_2_. The low intensity peak at 1735 cm^–1^ is due to carbonyl (C=O) stretching. The peaks at 1645 and 1548 cm^–1^ are attributed to the C=C of the aromatic ring structures present in the PC.^36^ Another low intensity band near 1384 cm^–1^ is associated with the N–O stretching vibration.^37^ The band at 1105 cm^–1^ is from the stretching vibration of −C–O–C. Additionally, the free O–H band was relatively weak compared to the H-bonded O–H band (Figure S8 in the Supporting Information). The absorption maximum at ∼3448 cm^–1^ was attributed to the four-coordinated water molecules; however, the absorption peak at 3749 cm^–1^ was associated with the three-coordinated water molecules within the water cluster. The broad absorption below 3600 cm^–1^ was identified as the hydrogen-bonded O–H stretching vibration, while the sharp band near 3700 cm^–1^ corresponded to the free (dangling) O–H stretching vibration. The presence of a free O–H band indicated the presence of three-coordinated water molecules, as four-coordinated water molecules do not have a free O–H band.^38^

**Figure 3:**
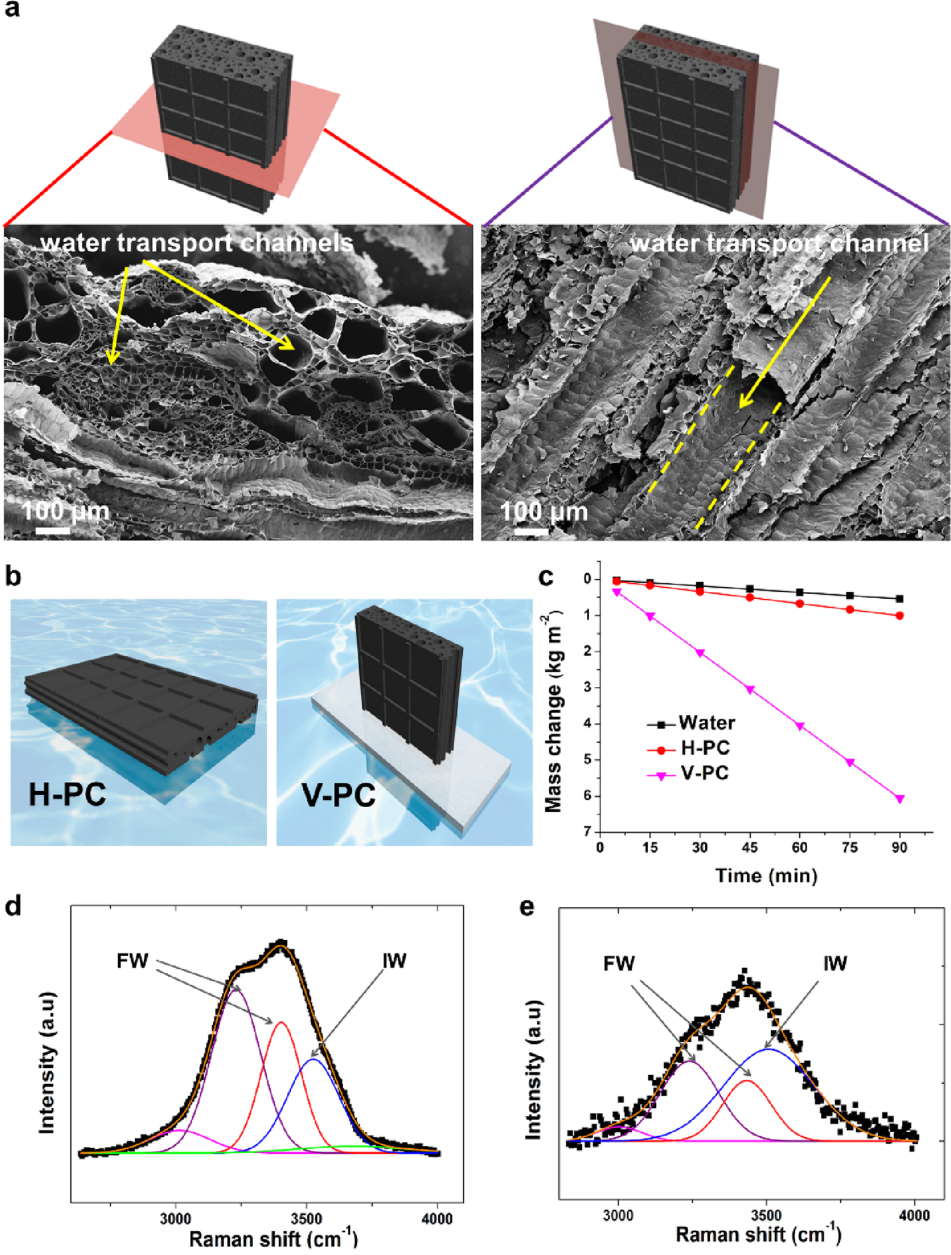
Orientation-dependent solar desalination using PC. (a) Cross-sectional SEM images of PC along the horizontal and vertical planes show the water diffusion channels. The dotted yellow lines highlight an air tube in PC. (b) Scheme of horizontally oriented PC (H-PC, left) and vertically oriented PC (V-PC) fixed by a piece of polystyrene foam in water (right). (c) Water mass change as a function of time for H-PC and V-PC, in comparison with bulk water evaporation without PC under 1 sun. Raman spectra of (d) bulk water and (e) water confined in PC. The Raman spectra are deconvoluted to differentiate the contributions from those of free water (FW) and intermediate water (IW).

Structurally, the PC contained long cylindrical pores derived from vascular bundles that extended through the entire stem, as shown by the transverse (Figure 3a) and longitudinal SEM images (Figure 3b). The long cylindrical pores are expected to be beneficial for solar desalination from two aspects. First, they facilitate water transport throughout PC. Second, they separate the confined water from bulk water to avoid heat loss during solar desalination. To visually demonstrate the water transport properties, PP and PC were placed in an equal amount of methylene blue solutions (Figure S9 in the Supporting Information). It took ∼8 h for the solution to reach halfway across the PP strip but only a few seconds to reach the top of PC (Video S1), demonstrating the necessity of complete pyrolysis instead of partial carbonization of biomaterials.^31,32,39−42^ Notably, after 7 min, most of the dye solution was absorbed by PC but not PP. The carbonized vascular bundles facilitated an efficient flow of fluid across PC (Video S2). The drastically improved hydrophilicity and water transport of PC, compared with PP, suggest that the former is an excellent material for solar water evaporation.

**Figure 4:**
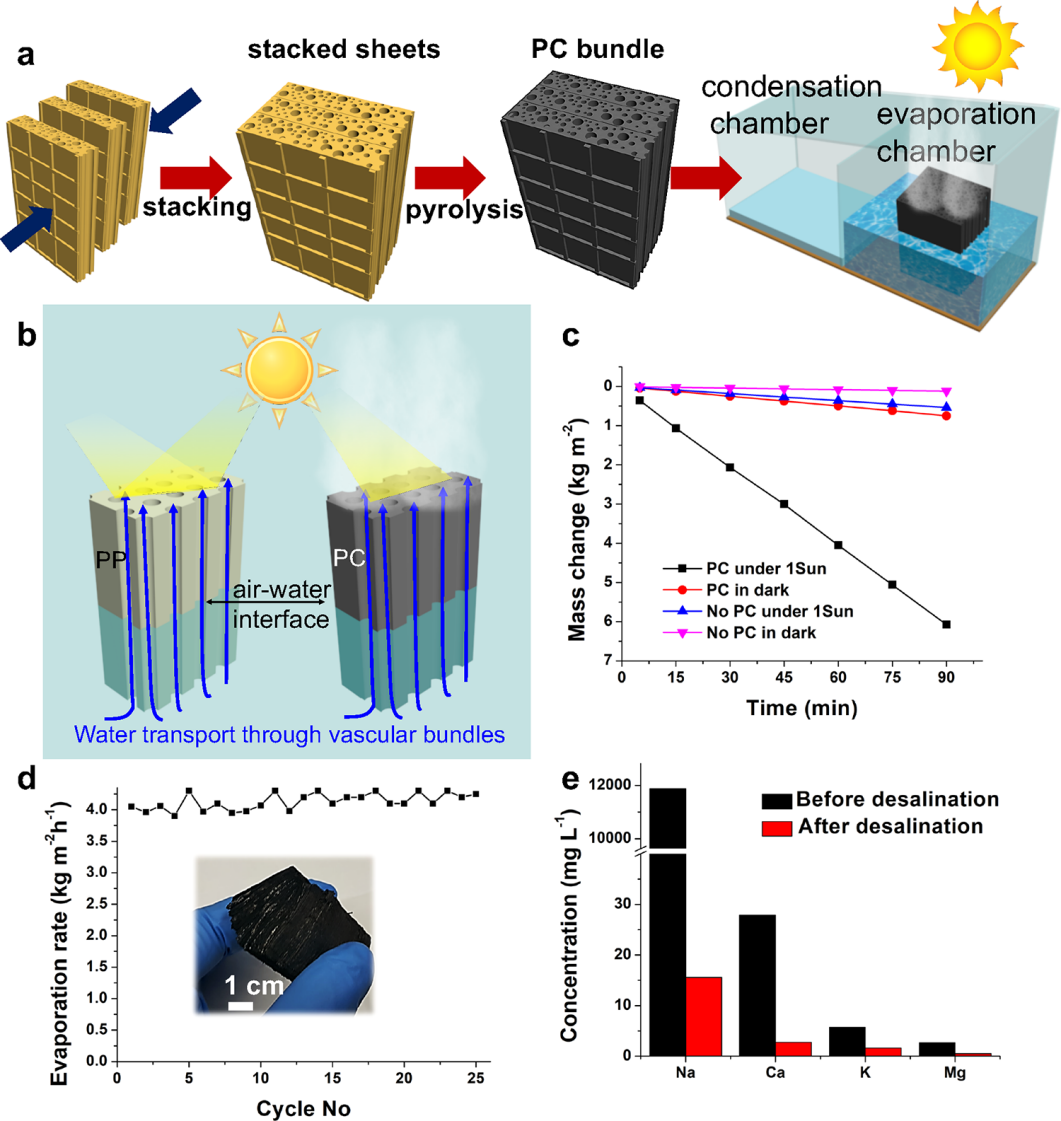
Performance of PC for solar desalination. (a) Schematic illustration of scaled-up PC bundles for solar desalination. (b) Highly efficient solar desalination by PC, because of its high solar light absorption, vascular transport of water, and steam generation on high surface areas of capillary pores. (c) Water mass changes over time with and without one sun illumination (labeled “under one sun” and “in dark,” respectively), with and without PC (labeled “PC” and “No PC,” respectively). (d) Evaporation rate over 25 cycles of reuse under one sun. (inset) Photograph of a PC bundle to illustrate that it can be handled easily. (e) Chemical analysis of saline water before and after desalination.

Due to efficient light absorption and well-designed structures for water transport and confinement, PC exhibited exceptional solar desalination performances. PC was tested for solar desalination of artificial seawater containing 3.5 wt % sea salt, which is the average salinity of ocean water.^43^ Because the long cylindrical pores served as pathways for transporting water to the upper ends of PC (Figure 3a), solar steam generation strongly depended on the orientation of the cylindrical pores in water. Under simulated solar irradiation (1 sun), the water evaporation rates were 0.67 and 4.1 kg m^2^ h^–1^, respectively, for PC with the cylindrical pores oriented horizontally (H-PC) and vertically (V-PC) to the water surface (see Figures 3b and 3c, as well as Figures S10 and S11 in the Supporting Information). This discrepancy was because the cylindrical pores of PC, when placed vertically on the water surface, facilitated water transport (Videos S2 and S3). Due to the surface tension of water, the mesopores and micropores induced a capillary effect and spontaneously pumped water up along the cylindrical pores. Since the height of water rising inside these pores was much larger than the thickness of PC (∼4 cm; see Figure S12 and Table S1 in the Supporting Information), water easily reached the top surface of V-PC. Additionally, as the vertical pores pumped water from the bottom to the upper surface, PC absorbed solar radiation and converted it to heat for steam generation (Figure 4b). On the upper surface, the open pores permitted steam to escape without any hindrance. The cylindrical pores also isolated water in PC from bulk water, thus decreasing the heat loss to the nonevaporative part of water.^31^ Additionally, the distribution of water states within the PC may indeed differ in pores oriented in various directions. The material exhibits similar pore sizes in both vertical and horizontal orientations, which is attributed to its composition with two transverse layers. The vertically oriented pores, exposed to solar illumination, show enhanced water transport due to continuous flow and increased evaporation rates. In contrast, the horizontal pores, shielded from direct solar exposure, exhibit lower water transport rates. This directional impact is a key consideration in understanding the confinement effects on water within PC material. The influence of gravity and solar exposure contributes to varied water states, emphasizing the nuanced behavior of PC in different orientations.

To understand the effects of vascular bundles on solar desalination, the vaporization enthalpy of PC-confined water was measured using differential scanning calorimetry (DSC) (see the Supporting Information). First, to validate the DSC method, we measured the vaporization enthalpy of bulk water. The experimental value, 2370 J g^–1^, was in good agreement (∼3% error) with the theoretical value of 2444 J g^–1^.^44^ However, the vaporization enthalpy for PC-confined water was only 1500 J g^–1^, ∼37% less than that of bulk water (Figure S13 in the Supporting Information). Additionally, the water vaporization enthalpy was estimated by comparing the spontaneous evaporation of bulk water with that of PC-confined water (Supporting Information). The equivalent water vaporization enthalpy (*E*_eq_) of PC-confined water was estimated to be 1092.7 J g^–1^, lower than the value determined by DSC. Nevertheless, both methods showed a decrease in the evaporation enthalpy of confined water, which is attributed to the confinement effect of PC porous networks. According to the water cluster theory,^38,45^ PC-confined water forms small clusters of a few to tens of water molecules, allowing single molecules to escape from the clusters at a lower energy cost than bulk water. Therefore, the evaporation enthalpy decreases.

We also used Raman analysis to corroborate the decreased vaporization enthalpy. Based on the strength of intermolecular hydrogen bonding, water is classified into three types: free water (FW), bound water, and intermediate water (IW).^45,46^ FW is associated with normal water–water hydrogen bonding; bound water molecules are attached to the PC surfaces; intermediately, IW corresponds to weakened water–water hydrogen bonding and is known as activated water. Thus, IW costs less energy to vaporize than FW and bound water.^15,46−48^ Raman spectra in the region of O—H stretching provided information about the type of hydrogen bonding in bulk water and PC-confined water (Figures 3d and 3e). For bulk water, the Raman peaks at 3015, 3230, and 3403 cm^–1^ corresponded to FW, and the peaks at 3525 and 3676 cm^–1^ were for IW (Figure 3d).^49^ For PC-confined water (Figure 3e), the peaks at 3002, 3240, and 3433 cm^–1^ corresponded to FW, and that at 3507 cm^–1^ was related to IW (weakly hydrogen-bonded). The peak-intensity ratio of IW to FW was 0.9 for PC-confined water, higher than the value of 0.3 for bulk water, suggesting that PC-confined water contained more IW than bulk water.

The enhanced ratio of IW to FW of PC also aligns with the elemental analysis (Table S2 in the Supporting Information). The elemental composition of PC revealed a mostly hydrophobic nature with a high carbon content (83.5%) and low hydrogen content (0.56%). However, the presence of N (1.32%), O (13.95%), and S (0.67%) also contributes to some degree of hydrophilicity (less than 20%). This unique structure of PC, with a combination of hydrophobic and hydrophilic properties, plays a crucial role in the formation of water clusters. The high hydrophobicity of the PC pores allows water molecules to weakly interact with the walls through van der Waals forces, leading to the formation of water clusters. At the same time, the presence of polarized groups triggers dipolar “hydrophilicity”, resulting in a strip of hydrophilic zone along the PC pores, offering sites for nucleation of water clusters on the PC surface.^50^

The presence of these polar groups implies a weak interaction between the PC and water, enhancing the potential for IW formation in PC^49^ (see the Supporting Information). The higher content of IW disrupted the hydrogen-bonding network, reduced the evaporation enthalpy, and consequently facilitated solar steam generation.

The evaporation efficiency (η) is defined as , where *m*, *h*_*lv*_, and *I* are the mass flux of vapor, vaporization enthalpy of water, and irradiation power density, respectively. Following the equation, the evaporation efficiencies were 150% and 109%, based on the DSC-measured enthalpy and equivalent enthalpy from spontaneous water evaporation, respectively. As shown in the characterization above, PC has efficient solar light absorption (optically), straight vertical bundlelike microchannels for fast water transportation (structurally), isolated heating of PC-confined water from the bulk water (thermally), and a high percentage of IW at the molecular level to reduce the energy cost of water evaporation (thermodynamically). In addition, because of the three-dimensional structure,^51^ PC can recover energy from the environment to supplement the energy supply. PC fully retains the intact vascular bundles in two orthogonal directions. This feature gives the advantage of (1) water evaporation from the side walls of PC, and (2) the capability of recovering heat from the environment to supplement the energy supply (see Figure S14 in the Supporting Information). Because water evaporation consumes heat and reduces the temperature of PC sidewalls to be less than the room temperature, then PC absorbs latent heat^5^ from the surroundings by conduction, radiation, and convection.^51^ Moreover, we performed an energy distribution analysis to identify the energy loss and the efficiency of PC (Supporting Information). Because of these merits, PC has acquired its exceptional higher-than-unit solar desalination efficiency in a single-stage device, similar to those in previous reports.^31,51,52^ Based on a recent study by Xu et al.,^53^ a 5-fold increase in energy efficiency and evaporation rate (up to 20.5 kg m^–2^ h^–1^) could be possible if the materials are used in a multistage device to recycle the heat released from water condensation.

Cost and scalability are essential for practically deployable solar desalination devices in underdeveloped Africa. First, papyrus plants are renewable and are naturally abundant in the region. Second, the processing of papyrus paper has a low cost. Third, papyrus paper is an industrially scalable commodity that can be massively produced and processed, in contrast with natural materials, such as mushroom and lotus seedpods, that are produced at a smaller scale. These three merits make our V-PC design economical and scalable. To demonstrate the scalability, individual sheets of papyrus paper were stacked together and subjected to pyrolysis to produce PC bundles of different thicknesses and geometrical surface areas (Figure 4a). To prove the principle, in an in-house-built desalination setup, the PC bundles were inserted into a polystyrene foam to keep them afloat and vertical to the water surface. The rough PC bundle surfaces induced multiscattering of the incident light, elongated the optical path length, and enabled efficient solar light absorption.^13^ Regardless of the geometrical surface areas (0.20, 0.25, 0.45, 0.60, 0.80, and 4.75 cm^2^) (Figure S15 in the Supporting Information), the PC bundles showed exceptional solar steam generation performance (Figure 4c). We limited the surface area to 4.75 cm^2^, because of the limited size of our tube furnace for heating our samples. With an evaporation rate of up to 4.1 kg m^–2^ h^–1^ under 1 sun (4.75 cm^2^), PC outperformed reported materials to date (Table S3 in the Supporting Information; to compare material characteristics instead of devices, the table does not include engineering systems and multistage designs that recycle heat from water recondensation). Even in darkness, the PC bundle showed an ultrafast evaporation rate of 0.5 kg m^–2^ h^–1^, which is ∼6.5 times higher than bulk water in darkness (0.077 kg m^–2^ h^–1^) and 1.4 times higher than bulk water under 1 sun (0.35 kg m^–2^ h^–1^). These results unequivocally confirmed the reduced vaporization enthalpy and enhanced solar steam generation, because of the unique structure of PC.

The reusability of V-PC bundles was tested by running the evaporation experiment over 25 cycles, with ∼30 min in each cycle. After each cycle, the sample was dried before reuse. V-PC bundles showed good and stable performance during all cycles. Yet, no structural degradation after the stability test (Figure 4d). Moreover, the PC displayed a stable performance upon prolonged use (see Figure S16 in the Supporting Information). The consistent performance and steady evaporation rate of PC indicate its potential as a reliable and effective solution for long-term solar desalination, with added antifouling capability. After desalination, the concentrations of the main ions (Na^+^, Ca^2+^, K^+^, and Mg^2+^) in seawater dropped below those of drinking water set by the World Health Organisation (WHO),^54^ as determined by inductively coupled plasma–mass spectroscopy (ICP-MS) (see Figure 4e). Importantly, the cylindrical pores of V-PC were immune to salt clogging, because their hydrophobic surfaces enabled any accumulated salt to easily dissolve in water. The microchannels of PC, filled with brine, allowed excess salt to return to the bulk water through diffusion and convection. Continuous water provision and effective backflow of salt are achieved, thereby enabling a distinctive capability for salt rejection.^55^ Additionally, due to the bimodal pore mechanism,^41^ the wide pore openings allowed large salt crystals to fall back into the water, further mitigating the salt clogging problem.

To demonstrate the versatility of our PC-based solar desalination system, we employed PC to purify water containing a mixture of heavy-metal ions, including Zn^2+^, Cd^2+^, Cu^2+^, Ni^2+^, and Pb^2+^. These ions are frequently present in industrial wastewater at high concentrations. Utilizing solar energy, the purification process significantly dropped the heavy metal ion concentrations below the WHO guidelines (see Supporting Information and Figure S17 in the Supporting Information).

## Conclusions

This work features an important understanding of Nature-designed systems and their use for solar desalination. The work builds upon Nature but extends the capability of vascular bundles from water transport and confinement to water vaporization. The orientation of the vascular-buffer-derived pores plays a key role. When the pores are vertical to the water surface, they effectively confine local water and facilitate water transport to the free surface for vaporization, enabling an evaporation rate up to 4.1 kg m^–2^ h^–1^ under 1 sun, higher than any other solar desalination materials in a single-stage device in the literature. The exceptional solar evaporation rate is attributed to multiple advantages of PC, including near-unit light absorption, straight water-transport pores, isolation of water within pores from bulk water to minimize heat loss, and a high content of activated IW in PC-confined water to facilitate steam generation, all of which are designed by nature and yet require an in-depth understanding, so that one can master them. The method is scalable and broadly applicable to other vascular systems. The materials show high stability, reusability, and sustainability for desalination and other technologies including ultrafiltration, capacitive deionization,^9^ and electricity generation.^56^

## Methods

### Materials

Papyrus paper (PP) sheets (52 cm × 36 cm) were purchased from Khan el-Khalili (Egypt). NaCl was purchased from Sigma–Aldrich (USA). All aqueous solutions were prepared using deionized water with a resistivity of 18.2 MΩ cm, prepared by a Millipore system (USA).

### Preparation of Papyrus Carbon (PC)

PP with a thickness of 0.15 mm was cut into pieces of ∼3 cm × 4 cm in area and then carbonized into papyrus carbon (PC). The carbonization process began by loading PP into an electric tube furnace (MTI Corp.) under a constant stream of N_2_ at a flow rate of 30 standard cubic centimeters per minute (sccm). The furnace was heated from room temperature to 800 °C in 30 min and then held at 800 °C for 2 h. After carbonization, the furnace was naturally cooled to room temperature. The thickness of the PC noticeably expanded to 1.4 mm. If needed, then the resultant PC was activated via KOH activation. Briefly, PC was soaked in a 1 M KOH aqueous solution for 8 h and then dried on a hot plate at 70 °C for 6 h. The KOH-impregnated PC was activated by heating at 800 °C for 1 h under an N_2_ flow of 30 sccm. To prepare PC bundles, several PP sheets were stacked together and then subjected to pyrolysis, following the same protocol as that used for the single PC sheet.

### Instrumentation and Characterization

Electron microscopy imaging was performed on a field-emission scanning electron microscopy (FESEM) system (Model LEO 1550, Zeiss). UV-vis reflectance and transmittance spectra were collected on a Cary 5000 UV-vis spectrometer (Agilent Technologies). CO_2_ and N_2_ sorption isotherms were recorded by using a 3Flex Pore Analyzer (Micromeritics Instrument Corporation). Surface areas were based on the N_2_-physisorption isotherms collected at 77 K and calculated by using the Brunauer–Emmett–Teller (BET) method. Prior to physisorption, all samples were heated stepwise at 90 °C for 60 min and then at 350 °C for 900 min under a N_2_ atmosphere to remove residual moisture and hydrocarbons. Differential scanning calorimetry (DSC) (Model 2500 Discovery series, TA Instruments) was used to evaluate the vaporization enthalpy of free water and water confined within the PC pores. The elemental analysis was performed using an Elementar Vario El cube elemental analyzer. The Fourier transform infrared (FT-IR) spectra were obtained using a Nexeus-Nicolite-640-MSA FT-IR spectrometer. Raman spectra were obtained using a Raman spectrophotometer (WI Tec alpha 500, 100× object lens) with a laser wavelength of 633 nm.

### Solar Steam Generation

Solar desalination was conducted using an Oriel Sol3ATM Class AAA solar simulator (Model 94083A, 8 in. × 8 in. beam size) at an intensity of 1 kW m^–2^ (1 sun). The weight change of water was measured using a microbalance (Mettler). PC sheets and bundles were loaded into a beaker filled with saltwater (NaCl concentration = 35 parts per thousand). The PC sheets were oriented either parallel or perpendicular to the water surface. Parallel orientation was achieved by simply floating PC on the water surface. Vertical orientation was achieved by inserting PC through the center of a piece of polystyrene foam to create a PC/foam assembly, which was then floated on the water surface. The polystyrene foam served as a supporting material to keep the PC floating on water. The hydrophobic polystyrene foam could not be wetted by water and was impermeable to water vapor. A typical solar vapor generation setup is presented in Figure S18 in the Supporting Information. For comparison, a blank experiment setup was tested side by side, which had the same amount of water, the same type of container, the same supporting foam, and under the same environment. To prevent fouling of the PC, a regular cleaning with deionized water was performed as a precautionary measure.

## Data Availability

The data that support the plots within this paper and other findings of this study are available from the corresponding author upon reasonable request.

## Supporting Information Available

Supplementary notes, supplementary Figures S1–S18, supplementary Tables S1–S3, and supplementary references (PDF)
Supplementary video 1 (MP4)
Supplementary video 2 (MP4)
Supplementary video 3 (MP4)
