# Supplementary material for: Vascular Bundle for Exceptional Water Confinement, Transport, and Evaporation
Source: ACS Mater Lett. 2024 Jan 12;6(2):602–10. doi: 10.1021/acsmaterialslett.3c01593 (PMC10848287; doi:10.1021/acsmaterialslett.3c01593)
Supplement: Supplementary file 1 — tz3c01593_si_001.pdf [file tz3c01593_si_001.pdf]

Supplementary Information

Vascular Bundle for Exceptional Water Confinement, Transport and Evaporation

El Said A. Nouh[1,4], Tianyu Liu[1], Zacary L. Croft,[1] Guoliang Liu*,[1,2,3]

[1]Department of Chemistry, [2]Macromolecules Innovation Institute, and [3]Department of Materials Science and Engineering, Virginia Tech, Blacksburg, Virginia 24061, United States. [4]Nuclear Materials Authority, P.O. 530 El Maadi, Cairo, Egypt.

* Corresponding Author: gliu1@vt.edu

S-1: Papyrus plant

Papyrus plants are abundantly grown in the Nile Delta region of Egypt (**Supplementary Fig. 1**). The stem has a triangular cross-section (**Supplementary Fig. 2**). Within the stem, there is a white pith composed of ground tissues that are made of parenchyma cells. The parenchyma cells stack together in a honeycomb-like network, forming vertical intercellular air spaces called aerenchyma. Aerenchyma runs through the length of the entire stem. In a cross-sectional view, the parenchyma cells appear to be circularly arranged around the air passages. The parenchyma cell walls are mainly cellulose and hemicellulose. Fibrous vascular bundles embedded in the parenchymous material run parallel to the air passages. These passages serve as channels to carry nutrition and water to the plant head. The vascular bundles also give the stem rigidity and support. The inner fibers are made of xylem cells which carry water and have lignified walls, and the outer fibers are made of phloem cells which carry food and have cellulose walls. The fibro-vascular bundles can be seen by the naked eye in papyrus sheets, forming a fibre network that is filled with the parenchymous material (**Supplementary Fig. 3**)[1, 2].

S-2: Papyrus paper sheets

In typical industrial processing, papyrus sheets are prepared from papyrus plants as follows[1, 2]. Papyrus plant stems are cut into manageable lengths. The rind is peeled off to expose the pith. The pith is sliced into longitudinal thin strips along one of its three flat sides. A series of these strips are laid on a board side by side to make the first layer. A second layer is laid over in the transverse direction. The two layers are pressed together and allowed to dry, forming papyrus paper.

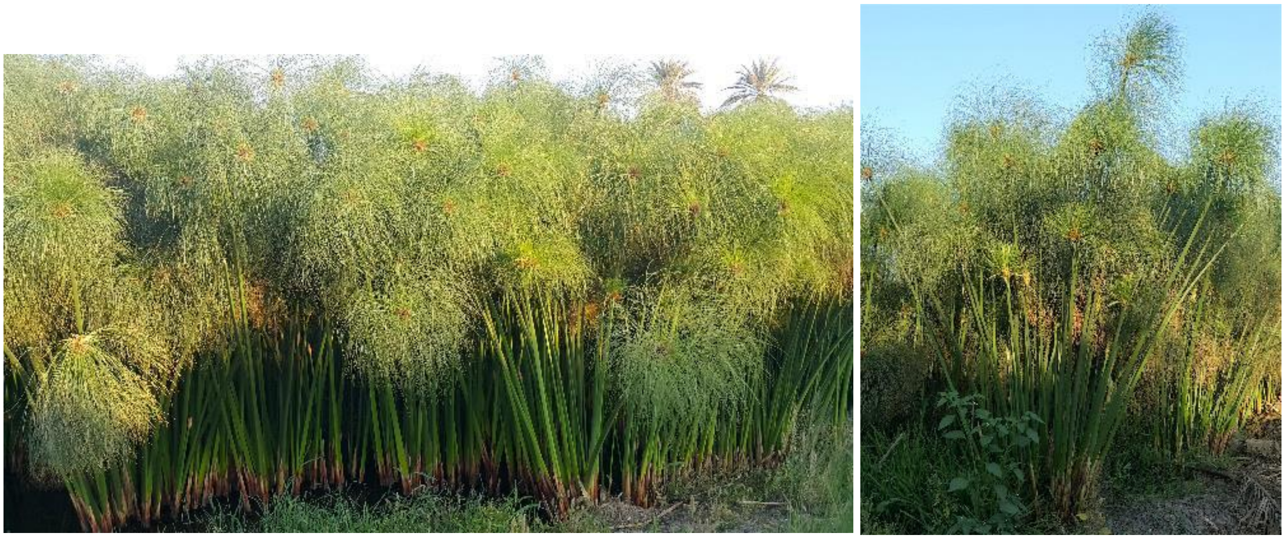

Supplementary Fig. 1. A Papyrus plant field in Egypt.

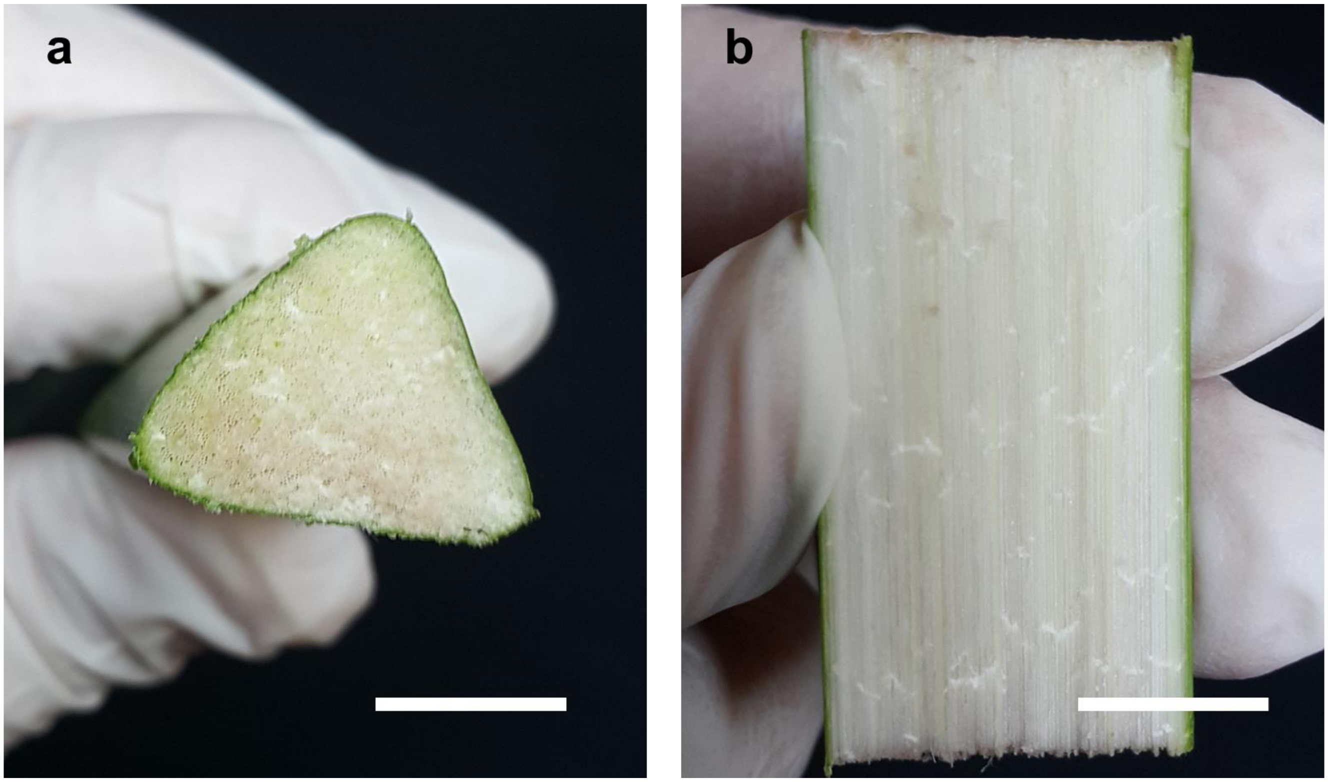

Supplementary Fig. 2. Cross-sectional photographs of a Papyrus stem along the (a) transverse and (b) longitudinal directions. The white vascular bundles are channels that allow water to flow in the stem. Scale bars: 1 cm.

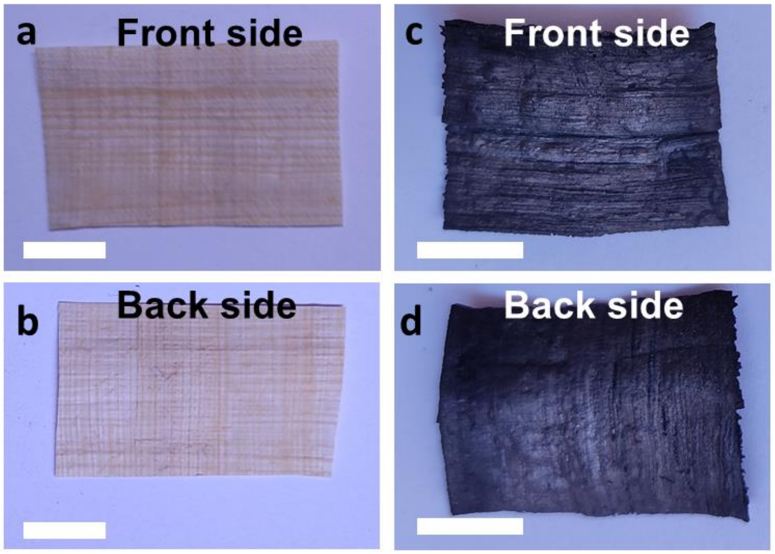

Supplementary Fig. 3. (a, b) PP shows the vascular bundles running vertically and horizontally, as visible to the naked eye. (c, d) The PC maintained the fabric lines running horizontally in the front side and vertically in the backside. Scale bars: 1 cm.

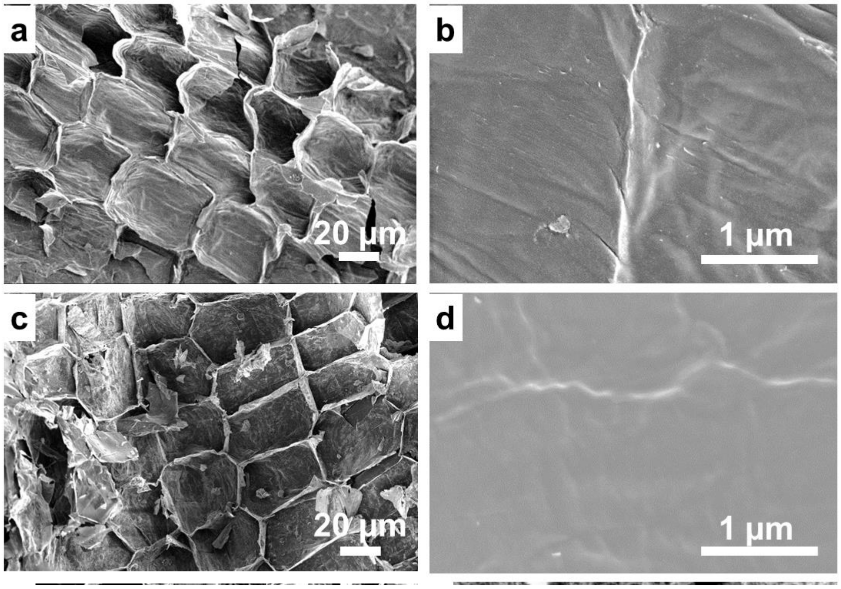

Supplementary Fig. 4. SEM images of (a, b) PP and (c, d) PC showing parenchyma cells forming a honeycomb-like network. The honeycomb-like network remains after pyrolysis. Inside each honeycomb, both (b) PP and (d) PC are devoid of pores.

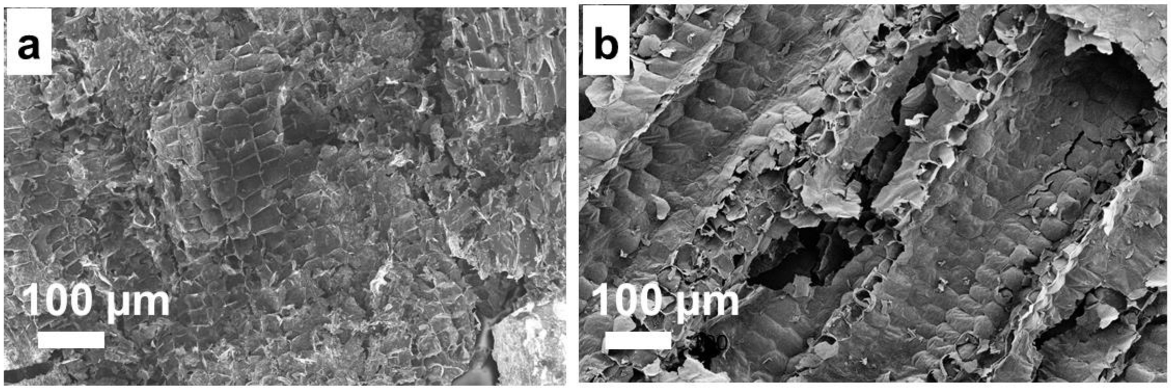

Supplementary Fig. 5. SEM of (a) PC exterior showing the parenchyma cells in a honeycomb-like network and (b) PC interior showing the honeycomb-like network building up channels for transporting water.

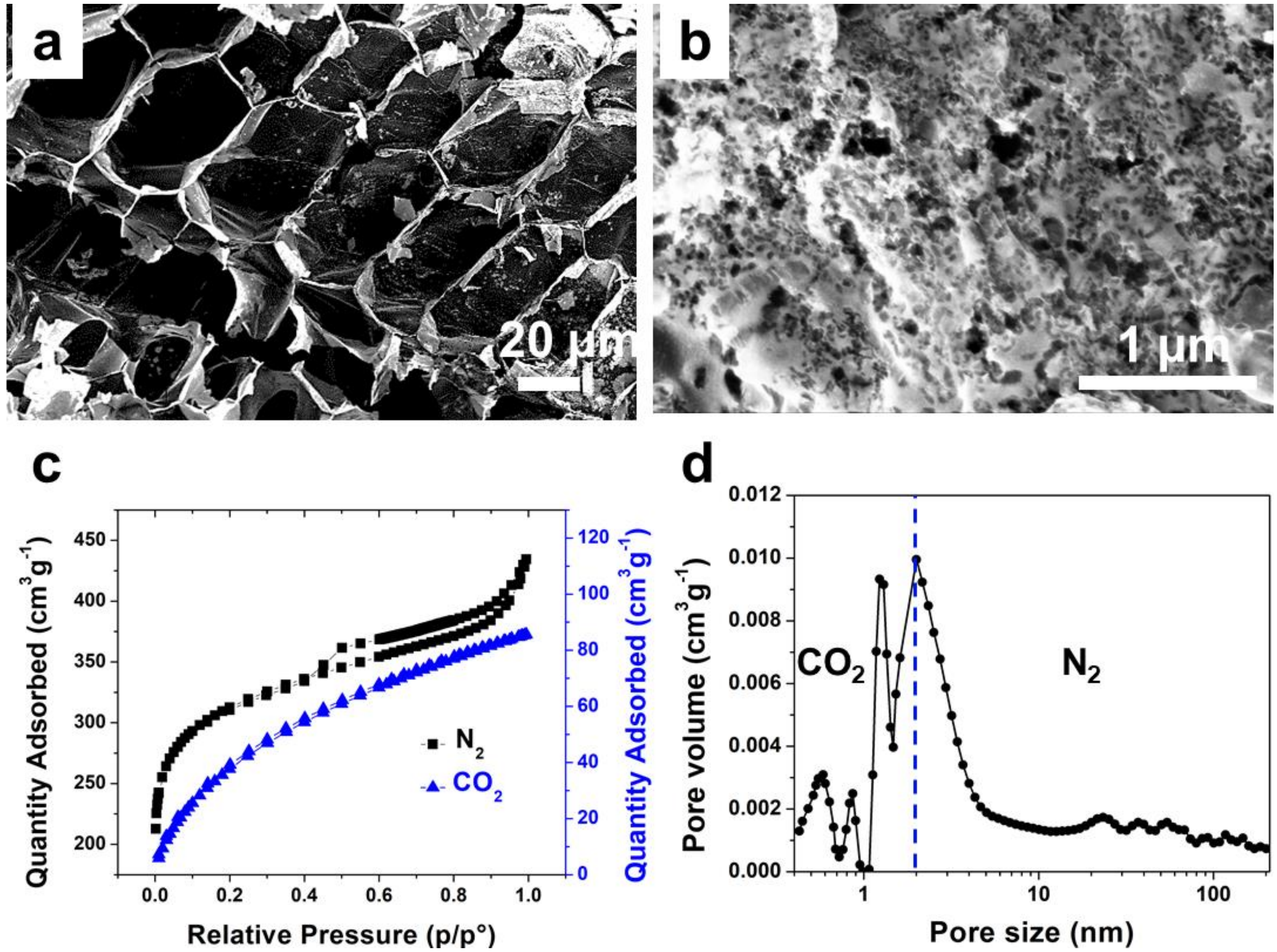

Supplementary Fig. 6. (a, b) SEM images, (c) CO_2- and N_2-physisorption isotherms, and (d) pore size distributions of activated papyrus carbon. After KOH activation, the surface of papyrus carbon became porous and the BET surface area increased to 1169 m^2 g^{-1}.

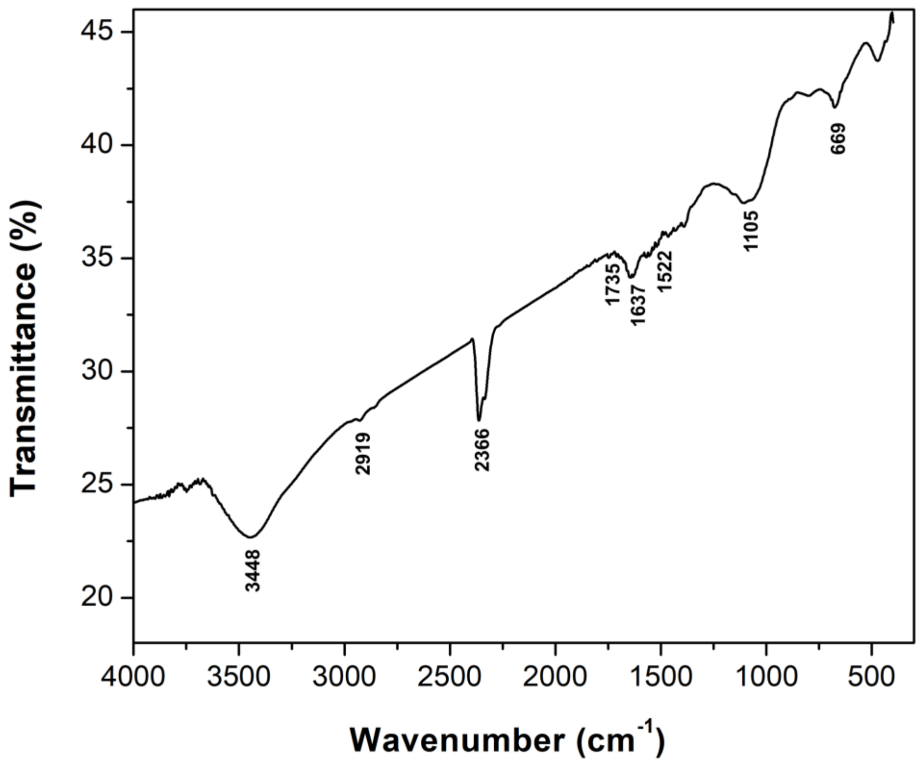

Supplementary Fig. 7. FT-IR spectra of PC.

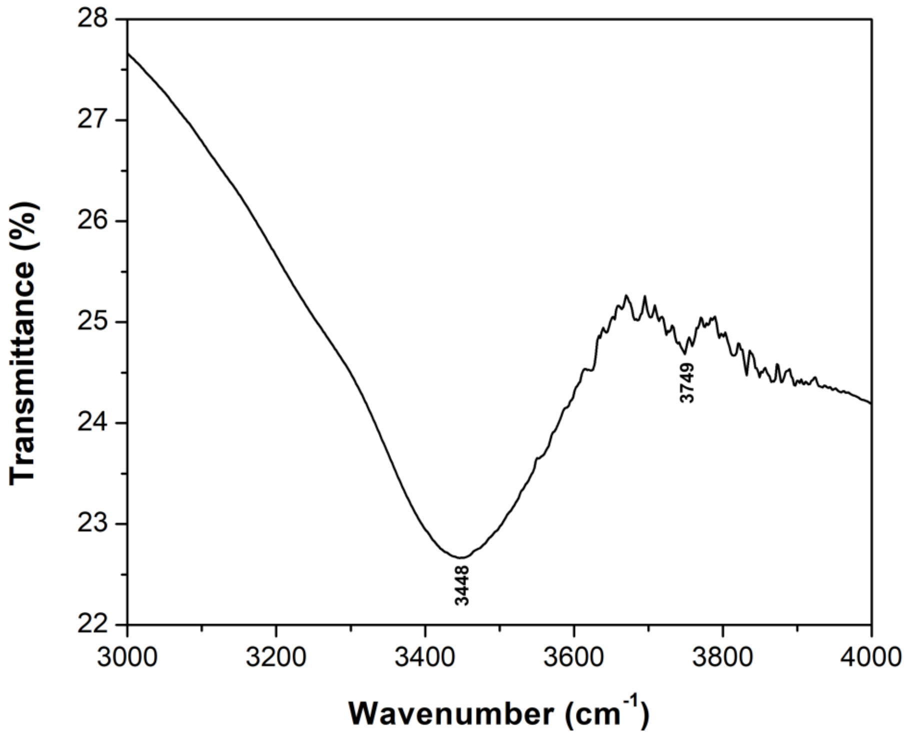

Supplementary Fig. 8. FT-IR spectra of PC-H_2O in the in H–bonded OH and free OH stretch region.

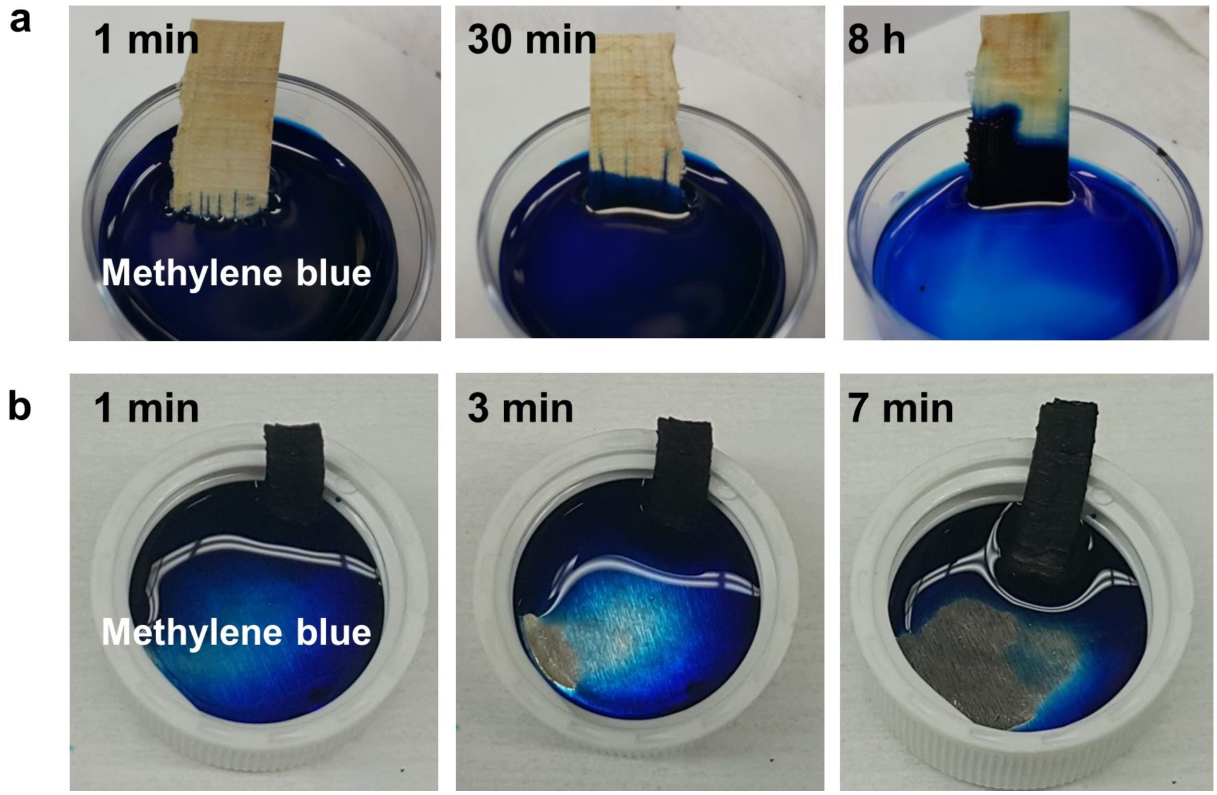

Supplementary Fig. 9. The capillary effect of microchannels in (a) PP and (b) PC. The photographs show that both PP and PC effectively soak up water, but PC has a much faster rate. For illustration purpose, methylene blue dye is added to the water.

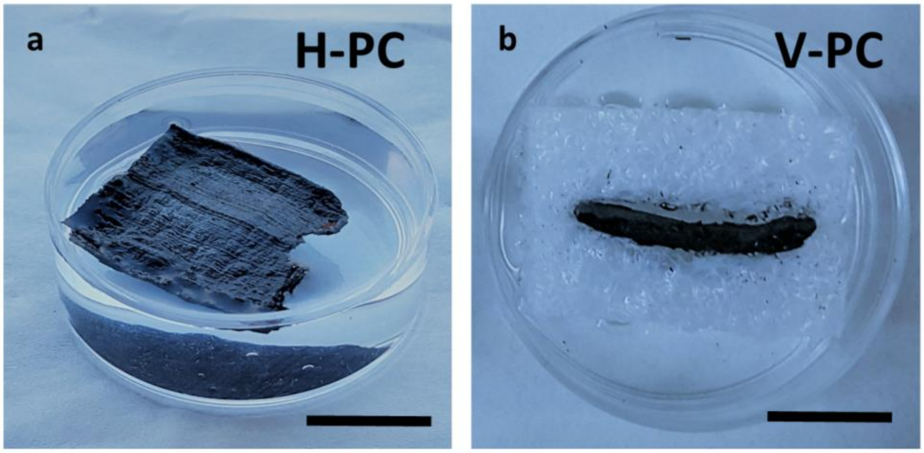

Supplementary Fig. 10. Photographs of a piece of PC (a) floating on water horizontally and (b) vertically. To ensure the vertical orientation, PC was embedded in a piece of polystyrene foam. Scale bars: 1 cm.

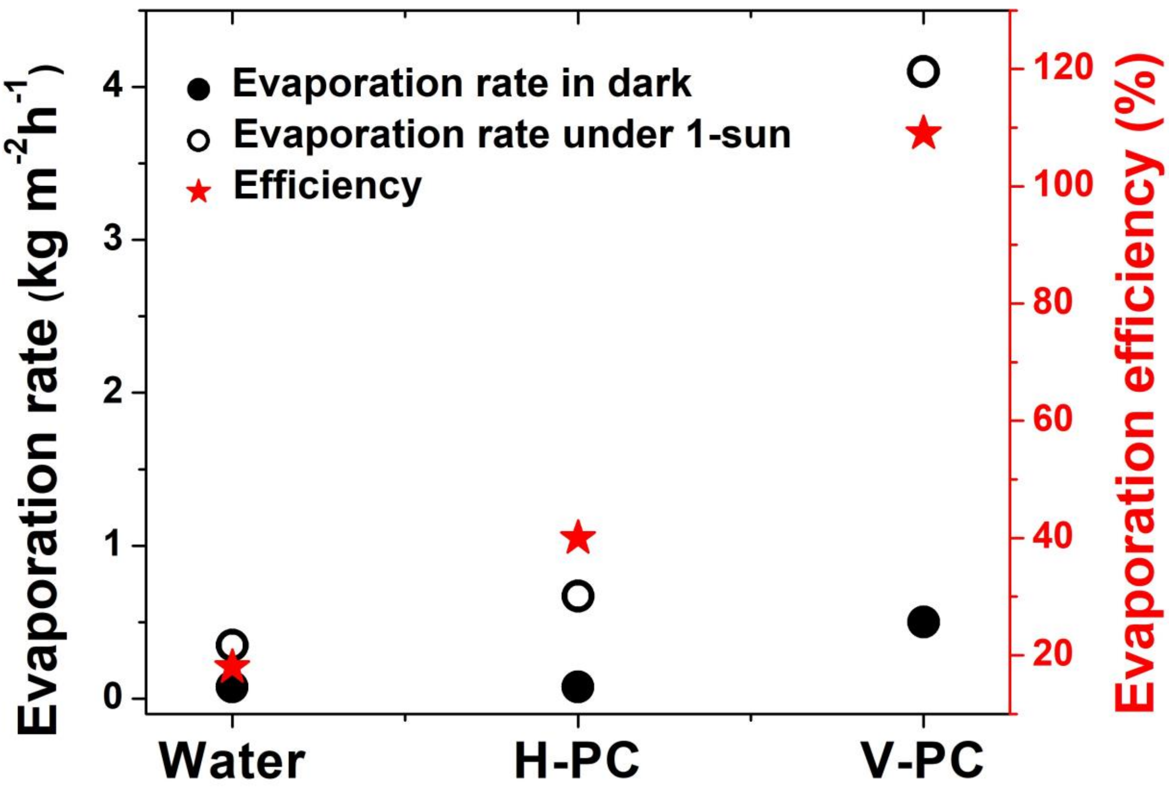

Supplementary Fig. 11. Evaporation rate in the dark and under 1 sun for bulk water, water confined in horizontally aligned PC (H-PC), and vertically aligned PC (V-PC).

S-3: Calculation of equilibrium height

The maximum height that water can rise inside PC is calculated following a previous report[3]. In a simplified model, inside two connected channels of different radii R_1 and R_2 (**Supplementary Fig. 12**), water must reach the same height according to the Jurin's Law[3-5]. The equilibrium height (h) is determined by:

$$h = \frac{2\gamma(R_1+R_2)}{\rho_w g(R_1^2+R_2^2)} \qquad (1)$$

where γ is the surface tension of water, ρ_w is the density of water, g is the gravity. Here we assume that the contact angel θ is negligible and approximates zero because PC is highly hydrophilic.

PC consists of capillaries with different sizes (**Supplementary Fig. 5 and 6**). The macropores in PC serve as artillery pumping channels for water. For simplicity, mesopores and micropores are not considered here for pumping water. Assume the macropores had different sizes of R_1, R_2, …, and R_k with the corresponding population of n_1, n_2, …, and n_k, thus, $\sum_{i=1}^{k} n_i = N$. The fraction of pores with a size R_i was $x_j=n_j/N$ (**Supplementary Table 1**). Therefore, a modified Eq. (1) can be used to calculate the height h as:

$$h = \frac{2\gamma \sum_{i=1}^{k} x_i R_i}{\rho_w g \sum_{\mathrm{i=1}}^{\mathrm{k}} \mathrm{x_i} R_i^2} \qquad (2)$$

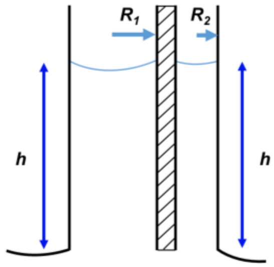

Supplementary Fig. 12. Schematic illustration of water height (h) inside papyrus carbon channels of varying pore sizes with radii of R_1 and R_2.

Supplementary Table 1. Macropore radii distribution in a representative cross-sectional area of PC.

R **(μm)**	**count (*n*$_i$)**	**$x_i = n_i/\sum n_i$**	***R*** **(μm)**	**count (*n*$_i$)**	**$x_i = n_i/\sum n_i$**
1	36	0.267	38	1	0.007
7	53	0.393	44	6	0.044
14	22	0.163	50	3	0.022
20	1	0.007	56	1	0.007
26	3	0.022	63	2	0.015
32	5	0.037	69	2	0.015

Substituting the values in **Supplementary Table 1** into Eq. (2), we obtained an equilibrium height (*h*) of 46.1 cm. Because the thickness of PC bundles is much smaller than 46.1 cm, water easily rises up inside the channels to reach the top of PC by the capillary effect.

S-4: Determination of water vaporization enthalpy by differential scanning calorimetry (DSC)

In the first method, water was heated in a DSC from 20 to 200 °C with a ramp rate of 5 °C min^{-1} under a nitrogen flow (20 mL min^{-1}). Bulk water showed a sharp peak, and the heat flux diminished after 100 °C, suggesting complete evaporation of bulk water. In contrast, PC-confined water showed different evaporation behaviors than bulk water (**Supplementary Fig. 13**). The DSC peak was much broader due to delayed evaporation of water bound to PC. Water evaporation was not yet completed right after the temperature reached 80 °C. The delayed water evaporation was due to the presence of bound water. The enthalpy of vaporization was calculated by integrating the area. Bulk water showed a vaporization enthalpy of 2370 J g^{-1}, in agreement with the reported experimental and theoretical values[6]. The vaporization enthalpy of PC-confined water, however, was only 1500 J g^{-1}, much less than that of bulk water. The reduced vaporization enthalpy is attributed to the confinement effect of the porous structures in PC.

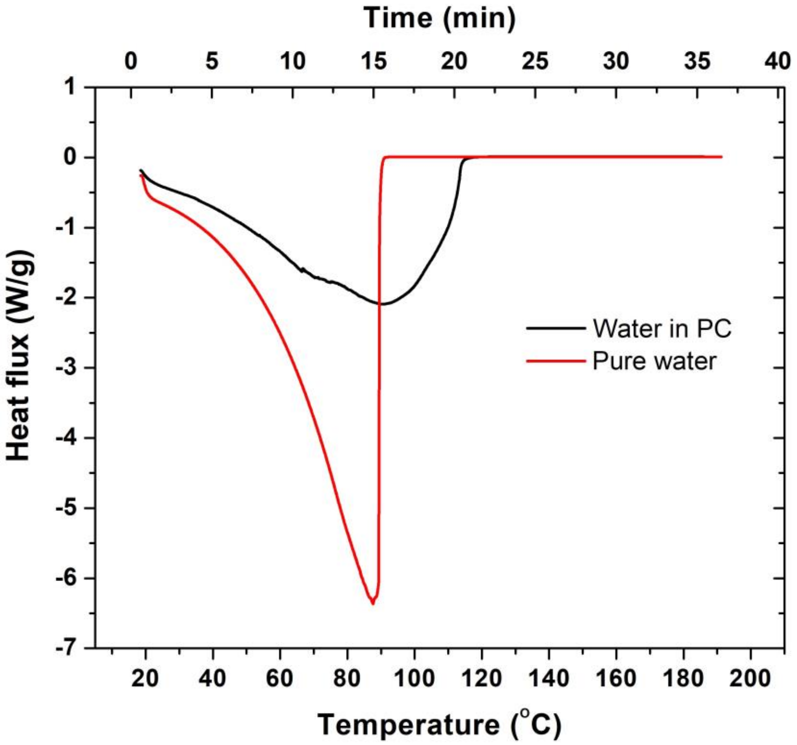

Supplementary Fig. 13. DSC traces of bulk water and PC-confined water, showing the heat flux as a function of temperature and time. Procedure segments: Equilibrate at 20 °C and then isothermal for 0.5 min; Afterward, ramp to 200 °C at a rate of 5 °C min^{-1}.

S-5: Determination of water vaporization enthalpy using equivalent water vaporization in the dark

Water evaporation is a natural process and occurs even in the absence of solar light[7]. It depends on many factors including atmospheric pressure, heat, air flow, and salinity[8]. Spontaneous vaporization under dark conditions is another method to evaluate the benefits of IW to solar vapor generation.

We determined the equivalent vaporization enthalpy by comparing the spontaneous evaporation of bulk water and PC-confined water in the dark, following a previous report[9]. During solar evaporation, PC is fully hydrated, so the energy associated with the evaporation of free and intermediate water should only be included in the real enthalpy of vaporization which consequently should be even lower.

The rates of evaporation of bare water and PC were examined. Under dark conditions, the total energy input (U_{in}) is supposed to be identical[9], thus the equivalent evaporation enthalpy of PC-confined water (E_{eq}) and the vaporization enthalpy of bulk water (theoretical value, E_o = 2444 J g^{-1})[9-11] must balance as follows,

$$U_{in} = E_0 m_0 = E_{eq} m_g \qquad (3)$$

where m_o and m_g are the mass changes of bulk water (as measured to be 71.9 mg) and PC-confined water (as measured to be 161 mg), respectively. The equivalent enthalpy was calculated to be 1092.7 J g^{-1}.

S-6: Calculation of solar vaporization efficiency

The obtained equivalent enthalpy values were used to calculate the evaporation efficiency (η),

$$\eta = \frac{m \times E_{eq}}{\mathrm{I}} \qquad (4)$$

where m is the vaporization rate after subtracting the vaporization rate in the dark and I is the power density of solar light (1 sun, 1000 W m^{-2}). For example, with a solar evaporation rate of 4.1 kg m^{-2} h^{-1} and E_{eq} of 1092.7 J g^{-1}, the efficiency was found to be 109%.

S-7: Intermediate water (IW) in the PC

Several factors affect the amount of IW:

1. The presence of functional groups in porous carbon: Specifically, functional groups that form hydrogen bonds with water increase the water adsorption and the amount of IW. Conversely, functional groups that form ionic interactions with water decrease water adsorption and the amount of IW. The

bound water, which strongly interacts with hydrophilic functional groups, is considered non-freezable water, while the IW and FW are freezable. The origin of the lack of crystallizability of water molecules absorbed in a material is different for hydrophilic and hydrophobic materials. For hydrophobic materials, the bound water is negligible, and the water molecules are randomly dispersed throughout the material matrix due to an entropy effect. Conversely, in hydrophilic materials, the water molecules are strongly bound to specific polar sites through hydrogen bonds, preventing them from aggregating to form crystalline water[12].

2. The size and shape of the pores in the porous carbon material can also impact the amount of IW. Pores that are large enough to accommodate water molecules and have a high specific surface area can result in a higher amount of IW.

3. The presence of defects or cracks in the porous carbon material can provide additional sites for water molecules to adsorb, further increasing the amount of IW.

The chemical composition of PC was determined using elemental analysis (EA) (**Supplementary Table 2**) and Fourier transform infrared (FTIR) spectroscopy (**Supplementary Fig. 7**). The FT-IR spectra of PC-H_2O indicating the H–bonded OH is presented in **Supplementary Fig. 8**. It is noteworthy that the PC contains groups showing its hydrophobic nature such as aromatic groups, in addition to a limited presence of polar group such as C=O and N–O, adding some hydrophilicity to the PC. The presence of the polar groups suggesting a weak interaction through hydrogen bonding between the PC and water, increasing IW in the PC.

Supplementary Table 2. Elemental analysis of PC

Element	wt %
Sulfur	0.67
Carbon	83.50
Hydrogen	0.56
Nitrogen	1.32
Oxygen*	13.95

* O% = 100%-C%-H%-N%-S%

S-8: Energy distribution analysis

Energy distribution analysis was used to evaluate the energy consumption and efficiency of PC.

The solar radiation intensity: 1 kW m^{-2}

The solar absorption efficiency of PC: 1 (100%)

The vaporization enthalpy: 1092 J g^{-1}

The energy efficiency: 109%

The amount of water desalinated: 4.1 kg m^{-2} h^{-1}

Let's assume the area of PC is 1 m^2

First, we need to calculate the amount of water desalinated per hour by multiplying the desalination rate by the area of the PC.

Amount of water desalinated per hour = 4.1 kg m^{-2} h^{-1} × 1 m^2 = 4.1 kg h^{-1}

To calculate the amount of energy required to vaporize this amount of water using the vaporization enthalpy:

Energy required for vaporization = 4.1 kg h^{-1} × 1092 J g^{-1} = 4.4772 × 106 J h^{-1} =1.24 kW

To calculate the energy input:

Energy input = (Solar radiation) x (Absorption efficiency) x (Area) = 1 kW m^{-2} × 1 × 1 m^2 = 1 kW

To calculate the energy loss using energy distribution analysis, we can use the following formula:

Energy loss = Energy input - Energy required = 1 kW – 1.24 kW = -0.24 kW

Therefore, 0.24 kW of energy are lost per unit area of PC.

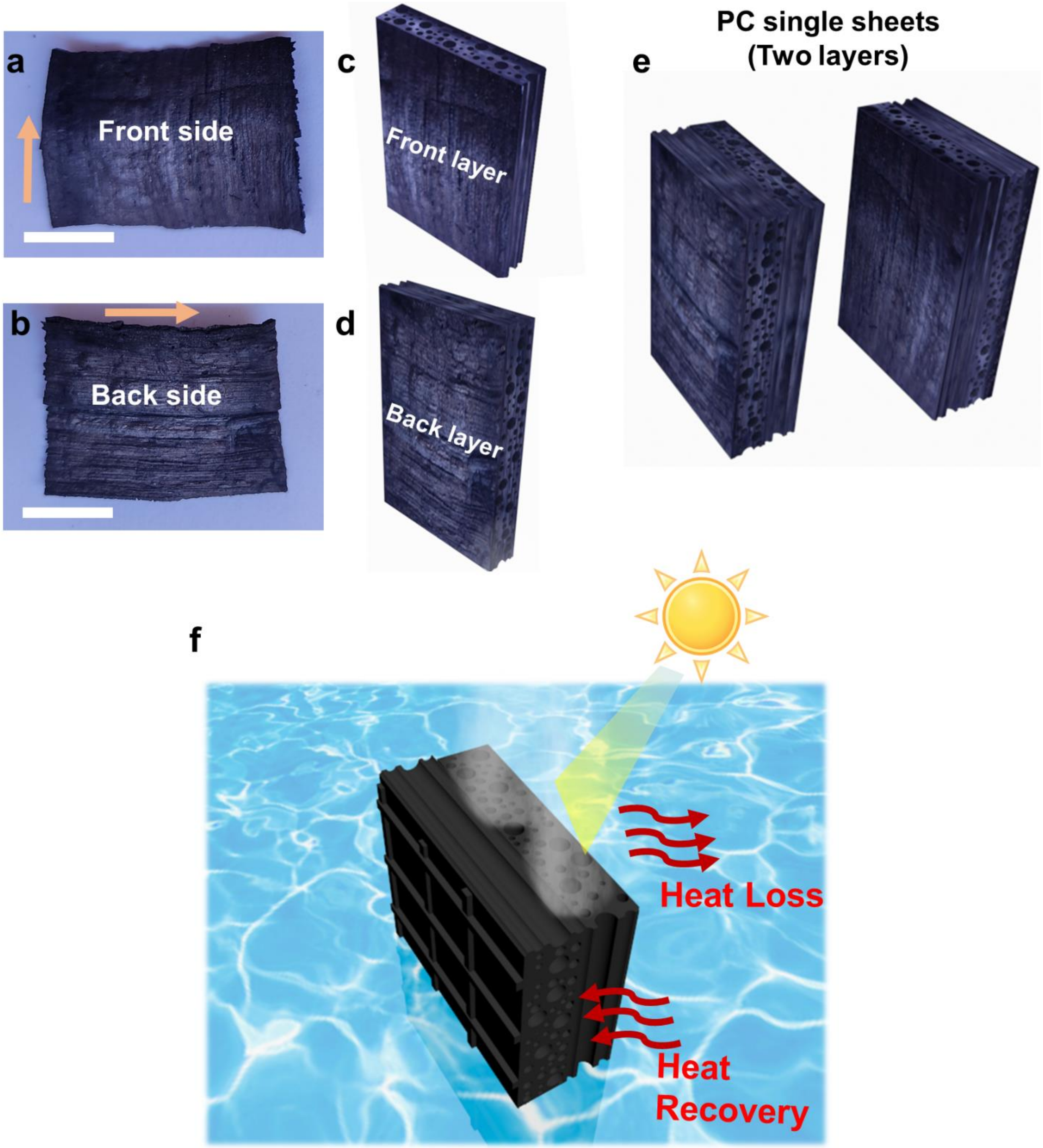

Supplementary Fig. 14. The PC maintained the fabric line running (a) vertically in the front side and (b) horizontally in the backside. (c,d) A piece of PC single sheet showing top and side pores of front and back layers, respectively. (e) PC single sheets showing the front and back layers united together with top and side pores. (f) A scheme of water evaporation in two different directions: Upward direction due to the sun light and sideward direction due to recovery of heat from the surroundings.

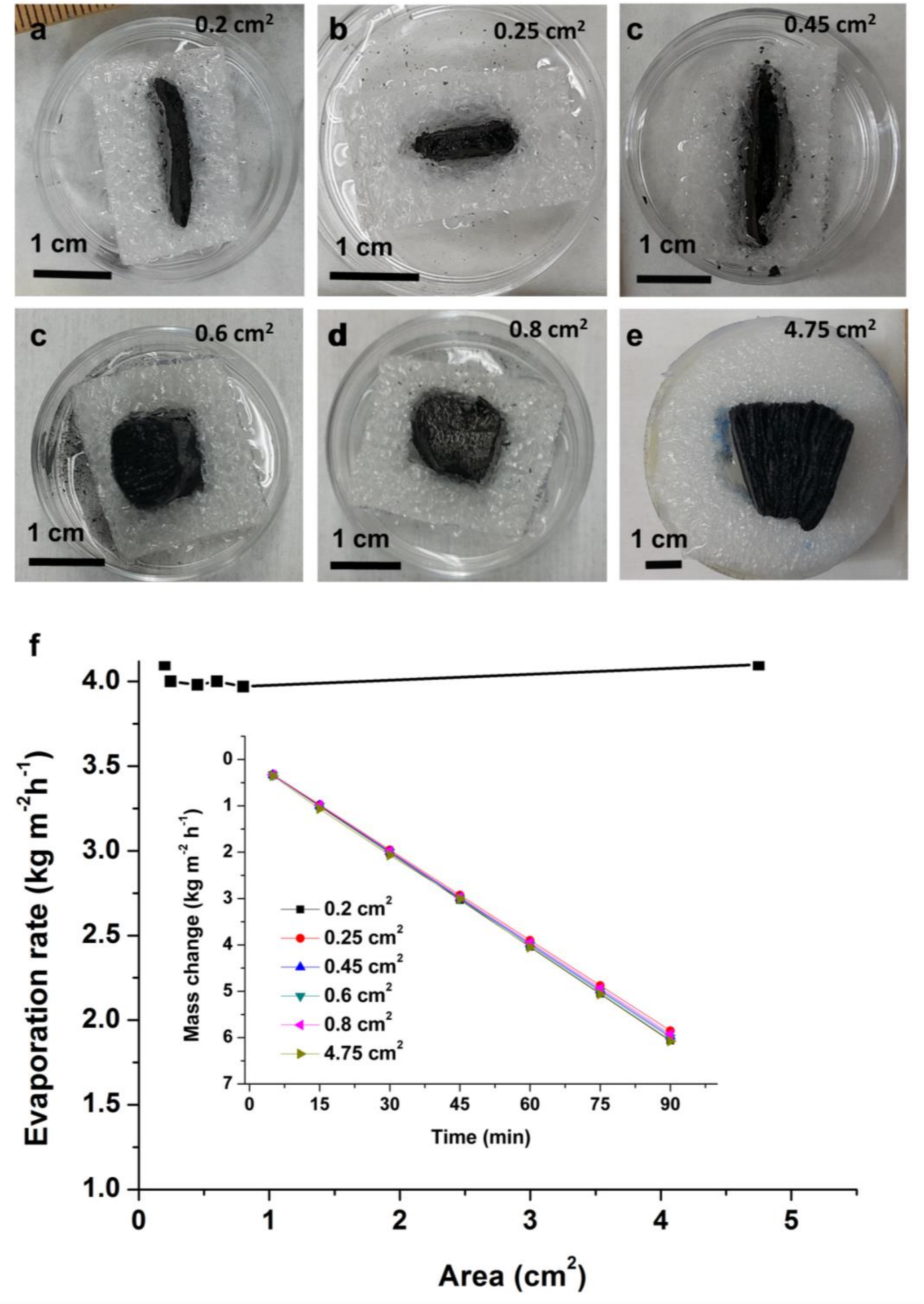

Supplementary Fig. 15. (a-e) Photographs and (f) solar vaporization rates of PC with different geometric surface areas. The inset shows the mass change over time for PC with different geometric surface areas.

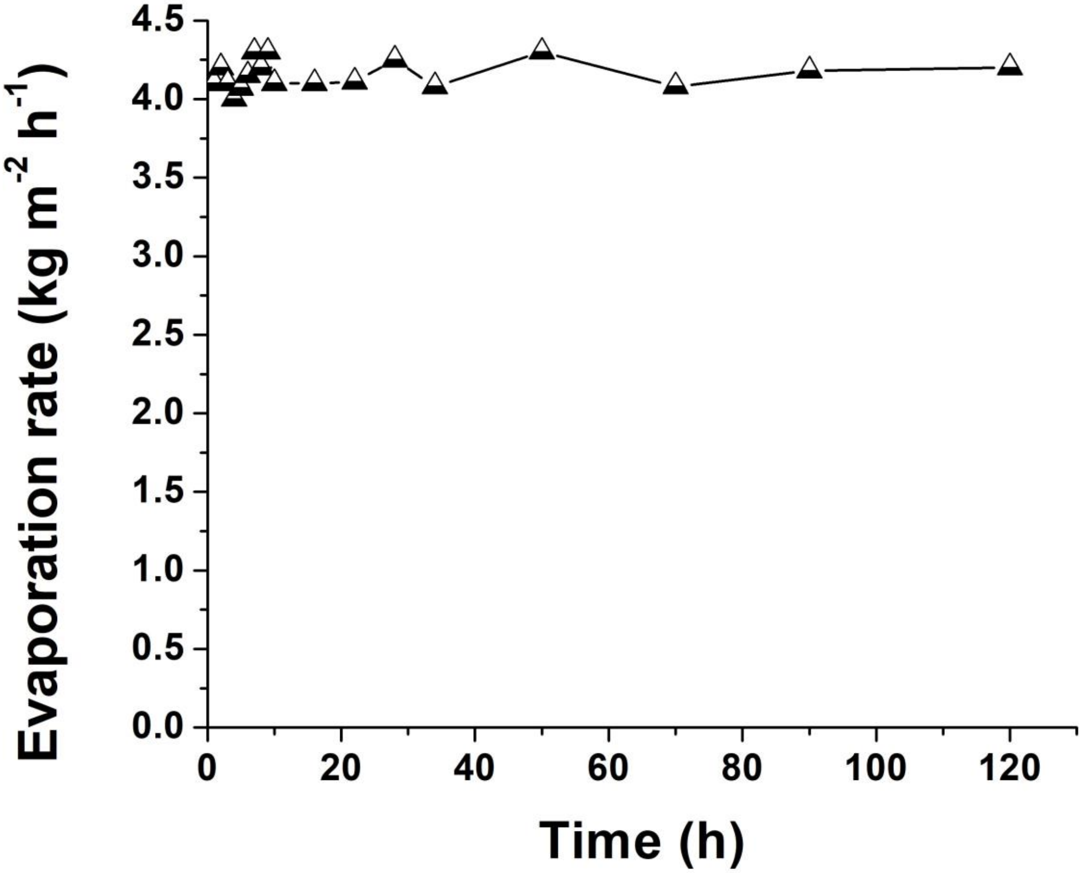

Supplementary Fig. 16. Evaporation rate of a re-used PC for 120 h under 1 sun.

Supplementary Table 3. Comparison of the water vaporization rates by PC and other materials. For meaningful comparison, we limit the materials to those tested under 1 kW m^{-2} (1 sun). Multistage engineering is not considered in testing these materials.

	Evaporation rate (kg m^{-2} h^{-1})	Efficiency (%)	Reference
carbonized mushrooms	1.475	78	13
porous graphene	1.50	80	14
Hierarchical nanowires coated copper foam	1.55	91	15
reduced graphene oxide–polyurethane nanocomposite foam	0.9	65	16
polypyrrole coated stainless steel mesh	0.92	58	17
bi-layered reduced graphene oxide film	1.31	83	18
hierarchical microstructured copper phosphate–PDMS composite sheets	1.01	63.6	19
carbonized bamboos	3.13	132	10
natural wood with a bilayer structure	1	57.3	3
graphene aerogel	0.75	53.6	20
carbon nanotube-modified flexible wood membrane	0.95	65	21
graphene oxide film	1.45	80	22
structure consisting of carbon foam and graphite layer	1	64	23
vertically aligned graphene sheets membrane	1.62	86.5	24
polydopamine filled bacterial nanocellulose hydrogel	1.13	78	25
hierarchical graphene foam	1.4	90	26
flame-treated wood	1.05	72	27
durable monolithic polymer foam	1.17	80.5	28
modified graphene aerogel	1.25	76.9	29
flexible thin film black gold membranes	0.47	42	30
carbon nanotube modified filter paper and a commercial Nafion	1.5	75	31
graphene oxide film	2.01	85	32
paper based reduced graphene oxide and porous insulation layer	1.778	80.6	33
reduced graphene oxide and 1D multi-walled carbon nanotubes	1.22	80.4	34
plasmonic absorber	1	63	35
black TiOx and stainless-steel mesh	0.8012	50.3	36
3D graphene	2.6 kg m^{-2} h^{-1} g^{-1}	87	37
Hydratable light absorbing hydrogel	3.6	92	9
carbon nanoparticles		24	38
aluminum nanoparticles	1	58	39
paper based AuNPs film	NR	77.8 *	40
polymer gel	3.2	94	41
bilayered hybrid biofoam graphene oxide	NR	83 **	42

floating solar receiver	4.4×10^{-4} kg m^{-2} s	NR	43
floating	NR	90	44
Contactless	NR	24.6	45
Carbon black	NR	60	46
Papyrus carbon	4.1	109	This work

NR: Not Reported
* under 4 sun
** under 10 sun

S-9: Heavy metal ions analysis in waste water samples

Supplementary Fig. 17 displays various heavy metal ion concentrations in water samples before and after solar desalination with PC. All the values after desalination fall significantly below the WHO guidelines. According to the WHO, the concentrations of copper, zinc, cadmium, nickel, and lead ions should below 2 mg L^{-1}, 3 mg L^{-1}, 0.003, 0.02, and 0.01 mg L^{-1}, respectively, for human health safety.

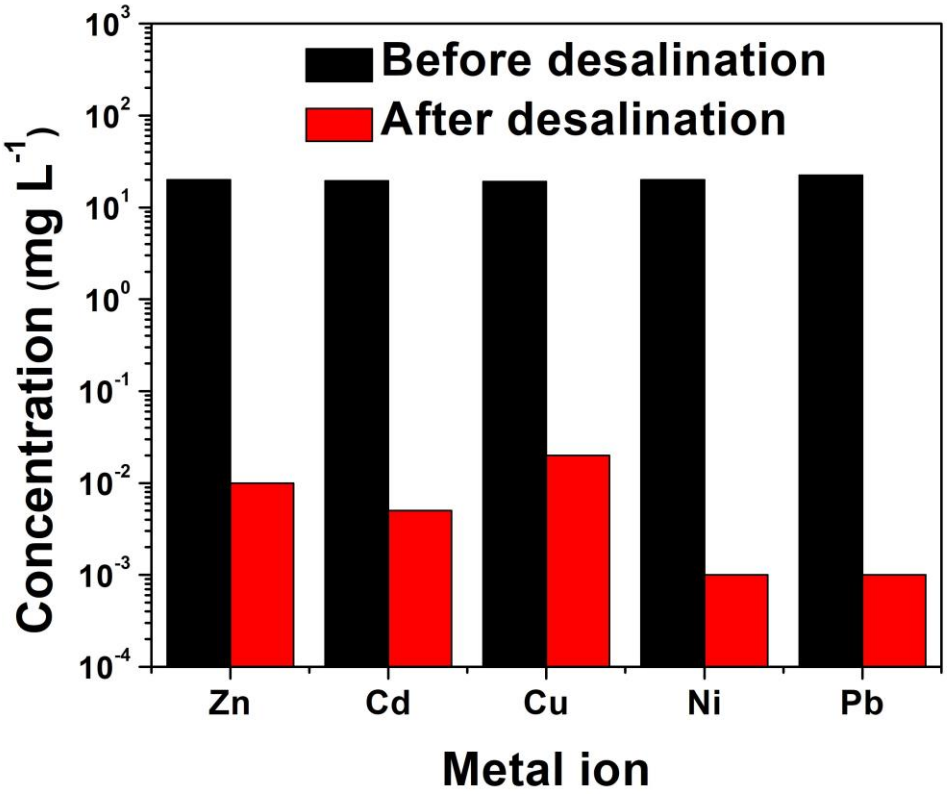

Supplementary Fig. 17. Concentration of heavy metal ions in the solution before and after purification using PC-solar desalination.

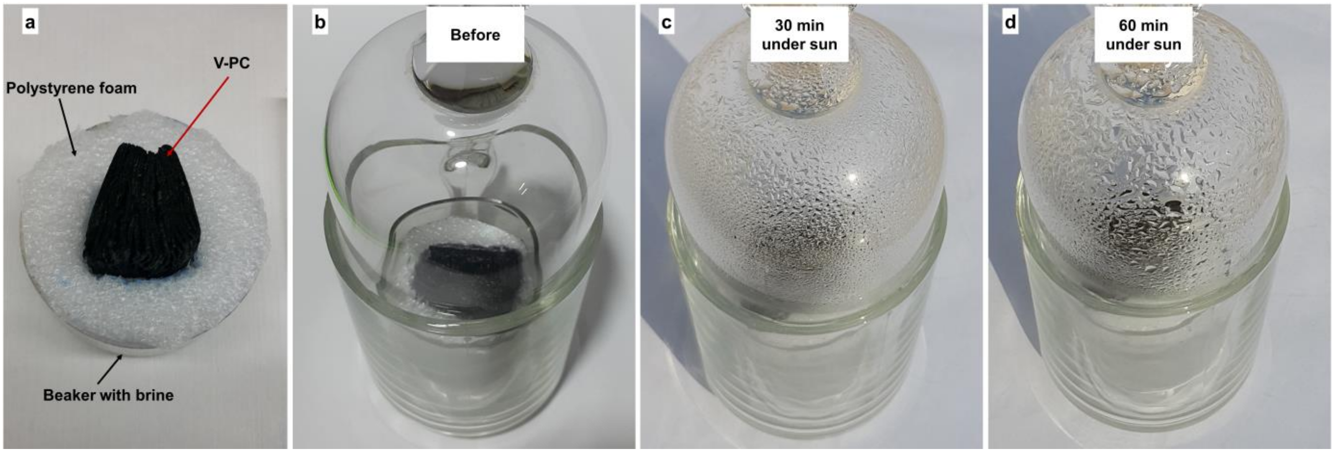

Supplementary Fig. 18. (a) A typical solar vapor generation set-up with PC. (b) Photograph depicting a prototype of a water collection device before light irradiation. (c, d) Images captured after exposure to solar light for different time intervals: (c) 30 min, (d) 60 min.

Supplementary Video 1: PC is highly effective and efficient in soaking up and transporting water from the bottom to the upper surface. Methylene blue dye is added for clear illustration.

Supplementary Video 2: Water droplets quickly spread over V-PC with unmeasurable contact angle. Water is quickly transported through the vascular bundle-derived pores, showing high water transport efficiency.

Supplementary Video3: Water droplets spread over H-PC with a measurable contact angle. Water can hardly transport through the vascular bundle-derived pores, nor the inter-fiber voids.

References

1. P. T. Nicholson, I. S., Ancient Egyptian Materials and Technology. *Cambridge University Press, UK* **2000**.
2. Wallert, A., The Reconstruction of Papyrus Manufacture: A Preliminary Investigation. *Studies in Conservation* **1989,** *34*, 1-8.
3. Zhu, M.; Li, Y.; Chen, G.; Jiang, F.; Yang, Z.; Luo, X.; Wang, Y.; Lacey, S. D.; Dai, J.; Wang, C.; Jia, C.; Wan, J.; Yao, Y.; Gong, A.; Yang, B.; Yu, Z.; Das, S.; Hu, L., Tree-Inspired Design for High-Efficiency Water Extraction. *Advanced Materials* **2017,** *29*, 1704107.
4. Das, S.; Mitra, S. K., Different regimes in vertical capillary filling. *Physical Review E* **2013,** *87*, 063005.
5. Quéré, D., Inertial capillarity. *Europhysics Letters (EPL)* **1997,** *39*, 533-538.
6. Barbara, B.; Massimiliano, B.; Michela, C.; Attilio, C., Water evaporation from gel beads. *Journal of Thermal Analysis and Calorimetry* **2010,** *103*, 81-88.
7. Penman, H. L., Natural evaporation from open water, bare soil and grass. *Proceedings of the Royal Society of London. Series A. Mathematical and Physical Sciences* **1948,** *193*, 120 - 145.
8. Song, H.; Liu, Y.; Liu, Z.; Singer, M. H.; Li, C.; Cheney, A. R.; Ji, D.; Zhou, L.; Zhang, N.; Zeng, X.; Bei, Z.; Yu, Z.; Jiang, S.; Gan, Q., Cold Vapor Generation beyond the Input Solar Energy Limit. *Advanced Science* **2018,** *5*, 1800222.
9. Zhou, X.; Zhao, F.; Guo, Y.; Rosenberger, B.; Yu, G., Architecting highly hydratable polymer networks to tune the water state for solar water purification. *Science Advances* **2019,** *5*, eaaw5484.

10. Bian, Y.; Du, Q.; Tang, K.; Shen, Y.; Hao, L.; Zhou, D.; Wang, X.; Xu, Z.; Zhang, H.; Zhao, L.; Zhu, S.; Ye, J.; Lu, H.; Yang, Y.; Zhang, R.; Zheng, Y.; Gu, S., Carbonized Bamboos as Excellent 3D Solar Vapor-Generation Devices. *Advanced Materials Technologies* **2019,** *4*, 1800593.
11. Zhao, H.-Y.; Zhou, J.; Yu, Z.-L.; Chen, L.-F.; Zhan, H.-J.; Zhu, H.-W.; Huang, J.; Shi, L.-A.; Yu, S.-H., Lotus-Inspired Evaporator with Janus Wettability and Bimodal Pores for Solar Steam Generation. *Cell Reports Physical Science* **2020,** *1*, 100074.
12. Ping, Z. H.; Nguyen, Q. T.; Chen, S. M.; Zhou, J. Q.; Ding, Y. D., States of water in different hydrophilic polymers — DSC and FTIR studies. *Polymer* **2001,** *42*, 8461-8467.
13. Xu, N.; Hu, X.; Xu, W.; Li, X.; Zhou, L.; Zhu, S.; Zhu, J., Mushrooms as Efficient Solar Steam-Generation Devices. *Advanced Materials* **2017,** *29*.
14. Ito, Y.; Tanabe, Y.; Han, J.; Fujita, T.; Tanigaki, K.; Chen, M., Multifunctional Porous Graphene for High-Efficiency Steam Generation by Heat Localization. *Advanced Materials* **2015,** *27*, 4302-4307.
15. Gao, X.; Ren, H.; Zhou, J.; Du, R.; Yin, C.; Liu, R.; Peng, H.; Tong, L.; Liu, Z.; Zhang, J., Synthesis of Hierarchical Graphdiyne-Based Architecture for Efficient Solar Steam Generation. *Chemistry of Materials* **2017,** *29*, 5777-5781.
16. Wang, G.; Fu, Y.; Guo, A.; Mei, T.; Wang, J.; Li, J.; Wang, X., Reduced Graphene Oxide–Polyurethane Nanocomposite Foam as a Reusable Photoreceiver for Efficient Solar Steam Generation. *Chemistry of Materials* **2017,** *29*, 5629-5635.
17. Zhang, L.; Tang, B.; Wu, J.; Li, R.; Wang, P., Hydrophobic Light-to-Heat Conversion Membranes with Self-Healing Ability for Interfacial Solar Heating. *Advanced Materials* **2015,** *27*, 4889-4894.
18. Shi, L.; Wang, Y.; Zhang, L.; Wang, P., Rational design of a bi-layered reduced graphene oxide film on polystyrene foam for solar-driven interfacial water evaporation. *Journal of Materials Chemistry A* **2017,** *5*, 16212-16219.
19. Hua, Z.; Li, B.; Li, L.; Yin, X.; Chen, K.; Wang, W., Designing a Novel Photothermal Material of Hierarchical Microstructured Copper Phosphate for Solar Evaporation Enhancement. *The Journal of Physical Chemistry C* **2017,** *121*, 60-69.
20. Fu, Y.; Wang, G.; Mei, T.; Li, J.; Wang, J.; Wang, X., Accessible Graphene Aerogel for Efficiently Harvesting Solar Energy. *ACS Sustainable Chemistry & Engineering* **2017,** *5*, 4665-4671.
21. Chen, C.; Li, Y.; Song, J.; Yang, Z.; Kuang, Y.; Hitz, E.; Jia, C.; Gong, A.; Jiang, F.; Zhu, J. J. Y.; Yang, B.; Xie, J.; Hu, L., Highly Flexible and Efficient Solar Steam Generation Device. *Advanced materials* **2017,** *29 30*.
22. Li, X.; Xu, W.; Tang, M.; Zhou, L.; Zhu, B.; Zhu, S.; Zhu, J., Graphene oxide-based efficient and scalable solar desalination under one sun with a confined 2D water path. *Proceedings of the National Academy of Sciences* **2016,** *113*, 13953.
23. Ghasemi, H.; Ni, G.; Marconnet, A. M.; Loomis, J.; Yerci, S.; Miljkovic, N.; Chen, G., Solar steam generation by heat localization. *Nature Communications* **2014,** *5*, 4449.
24. Zhang, P.; Li, J.; Lv, L.; Zhao, Y.; Qu, L., Vertically Aligned Graphene Sheets Membrane for Highly Efficient Solar Thermal Generation of Clean Water. *ACS Nano* **2017,** *11*, 5087-5093.
25. Jiang, Q.; Gholami Derami, H.; Ghim, D.; Cao, S.; Jun, Y.-S.; Singamaneni, S., Polydopamine-filled bacterial nanocellulose as a biodegradable interfacial photothermal evaporator for highly efficient solar steam generation. *Journal of Materials Chemistry A* **2017,** *5*, 18397-18402.
26. Ren, H.; Tang, M.; Guan, B.; Wang, K.; Yang, J.; Wang, F.; Wang, M.; Shan, J.; Chen, Z.; Wei, D.; Peng, H.; Liu, Z., Hierarchical Graphene Foam for Efficient Omnidirectional Solar–Thermal Energy Conversion. *Advanced Materials* **2017,** *29*, 1702590.
27. Xue, G.; Liu, K.; Chen, Q.; Yang, P.; Li, J.; Ding, T.; Duan, J.; Qi, B.; Zhou, J., Robust and Low-Cost Flame-Treated Wood for High-Performance Solar Steam Generation. *ACS Applied Materials & Interfaces* **2017,** *9*, 15052-15057.
28. Chen, Q.; Pei, Z.; Xu, Y.; Li, Z.; Yang, Y.; Wei, Y.; Ji, Y., A durable monolithic polymer foam for efficient solar steam generation. *Chem Sci* **2018,** *9*, 623-628.
29. Fu, Y.; Wang, G.; Ming, X.; Liu, X.; Hou, B.; Mei, T.; Li, J.; Wang, J.; Wang, X., Oxygen plasma treated graphene aerogel as a solar absorber for rapid and efficient solar steam generation. *Carbon* **2018,** *130*, 250-256.

30. Bae, K.; Kang, G.; Cho, S. K.; Park, W.; Kim, K.; Padilla, W. J., Flexible thin-film black gold membranes with ultrabroadband plasmonic nanofocusing for efficient solar vapour generation. *Nature Communications* **2015,** *6*, 10103.
31. Yang, P.; Liu, K.; Chen, Q.; Li, J.; Duan, J.; Xue, G.; Xu, Z.; Xie, W.; Zhou, J., Solar-driven simultaneous steam production and electricity generation from salinity. *Energy & Environmental Science* **2017,** *10*, 1923-1927.
32. Li, X.; Lin, R.; Ni, G.; Xu, N.; Hu, X.; Zhu, B.; Lv, G.; Li, J.; Zhu, S.; Zhu, J., Three-dimensional artificial transpiration for efficient solar waste-water treatment. *National Science Review* **2017,** *5*, 70-77.
33. Wang, Z.; Ye, Q.; Liang, X.; Xu, J.; Chang, C.; Song, C.; Shang, W.; Wu, J.; Tao, P.; Deng, T., Paper-based membranes on silicone floaters for efficient and fast solar-driven interfacial evaporation under one sun. *Journal of Materials Chemistry A* **2017,** *5*, 16359-16368.
34. Wang, Y.; Wang, C.; Song, X.; Megarajan, S. K.; Jiang, H., A facile nanocomposite strategy to fabricate a rGO–MWCNT photothermal layer for efficient water evaporation. *Journal of Materials Chemistry A* **2018,** *6*, 963-971.
35. Zhou, L.; Tan, Y.; Ji, D.; Zhu, B.; Zhang, P.; Xu, J.; Gan, Q.; Yu, Z.; Zhu, J., Self-assembly of highly efficient, broadband plasmonic absorbers for solar steam generation. *Science Advances* **2016,** *2*, e1501227.
36. Ye, M.; Jia, J.; Wu, Z.; Qian, C.; Chen, R.; O'Brien, P. G.; Sun, W.; Dong, Y.; Ozin, G. A., Synthesis of Black TiOx Nanoparticles by Mg Reduction of TiO2 Nanocrystals and their Application for Solar Water Evaporation. *Advanced Energy Materials* **2017,** *7*, 1601811.
37. Yang, Y.; Zhao, R.; Zhang, T.; Zhao, K.; Xiao, P.; Ma, Y.; Ajayan, P. M.; Shi, G.; Chen, Y., Graphene-Based Standalone Solar Energy Converter for Water Desalination and Purification. *ACS Nano* **2018,** *12*, 829-835.
38. Neumann, O.; Urban, A. S.; Day, J.; Lal, S.; Nordlander, P.; Halas, N. J., Solar Vapor Generation Enabled by Nanoparticles. *ACS Nano* **2013,** *7*, 42-49.
39. Zhou, L.; Tan, Y.; Wang, J.; Xu, W.; Yuan, Y.; Cai, W.; Zhu, S.; Zhu, J., 3D self-assembly of aluminium nanoparticles for plasmon-enhanced solar desalination. *Nature Photonics* **2016,** *10*, 393-398.
40. Liu, Y.; Yu, S.; Feng, R.; Bernard, A.; Liu, Y.; Zhang, Y.; Duan, H.; Shang, W.; Tao, P.; Song, C.; Deng, T., A Bioinspired, Reusable, Paper-Based System for High-Performance Large-Scale Evaporation. *Advanced Materials* **2015,** *27*, 2768-2774.
41. Zhao, F.; Zhou, X.; Shi, Y.; Qian, X.; Alexander, M.; Zhao, X.; Mendez, S.; Yang, R.; Qu, L.; Yu, G., Highly efficient solar vapour generation via hierarchically nanostructured gels. *Nature Nanotechnology* **2018,** *13*, 489-495.
42. Jiang, Q.; Tian, L.; Liu, K. K.; Tadepalli, S.; Raliya, R.; Biswas, P.; Naik, R. R.; Singamaneni, S., Bilayered Biofoam for Highly Efficient Solar Steam Generation. *Adv Mater* **2016,** *28*, 9400-9407.
43. Ni, G.; Li, G.; Boriskina, Svetlana V.; Li, H.; Yang, W.; Zhang, T.; Chen, G., Steam generation under one sun enabled by a floating structure with thermal concentration. *Nature Energy* **2016,** *1*, 16126.
44. Tao, P.; Ni, G.; Song, C.; Shang, W.; Wu, J.; Zhu, J.; Chen, G.; Deng, T., Solar-driven interfacial evaporation. *Nature Energy* **2018,** *3*, 1031-1041.
45. Cooper, T. A.; Zandavi, S. H.; Ni, G. W.; Tsurimaki, Y.; Huang, Y.; Boriskina, S. V.; Chen, G., Contactless steam generation and superheating under one sun illumination. *Nature Communications* **2018,** *9*, 5086.
46. Dongare, P. D.; Alabastri, A.; Neumann, O.; Nordlander, P.; Halas, N. J., Solar thermal desalination as a nonlinear optical process. *Proc Natl Acad Sci U S A* **2019,** *116*, 13182-13187.
